# Supplementary figures and images for: Interspike intervals within retinal spike bursts combinatorially encode multiple stimulus features
Source: PLoS Comput Biol. 2020 Nov 6;16(11):e1007726. doi: 10.1371/journal.pcbi.1007726 (PMC7738174; doi:10.1371/journal.pcbi.1007726)

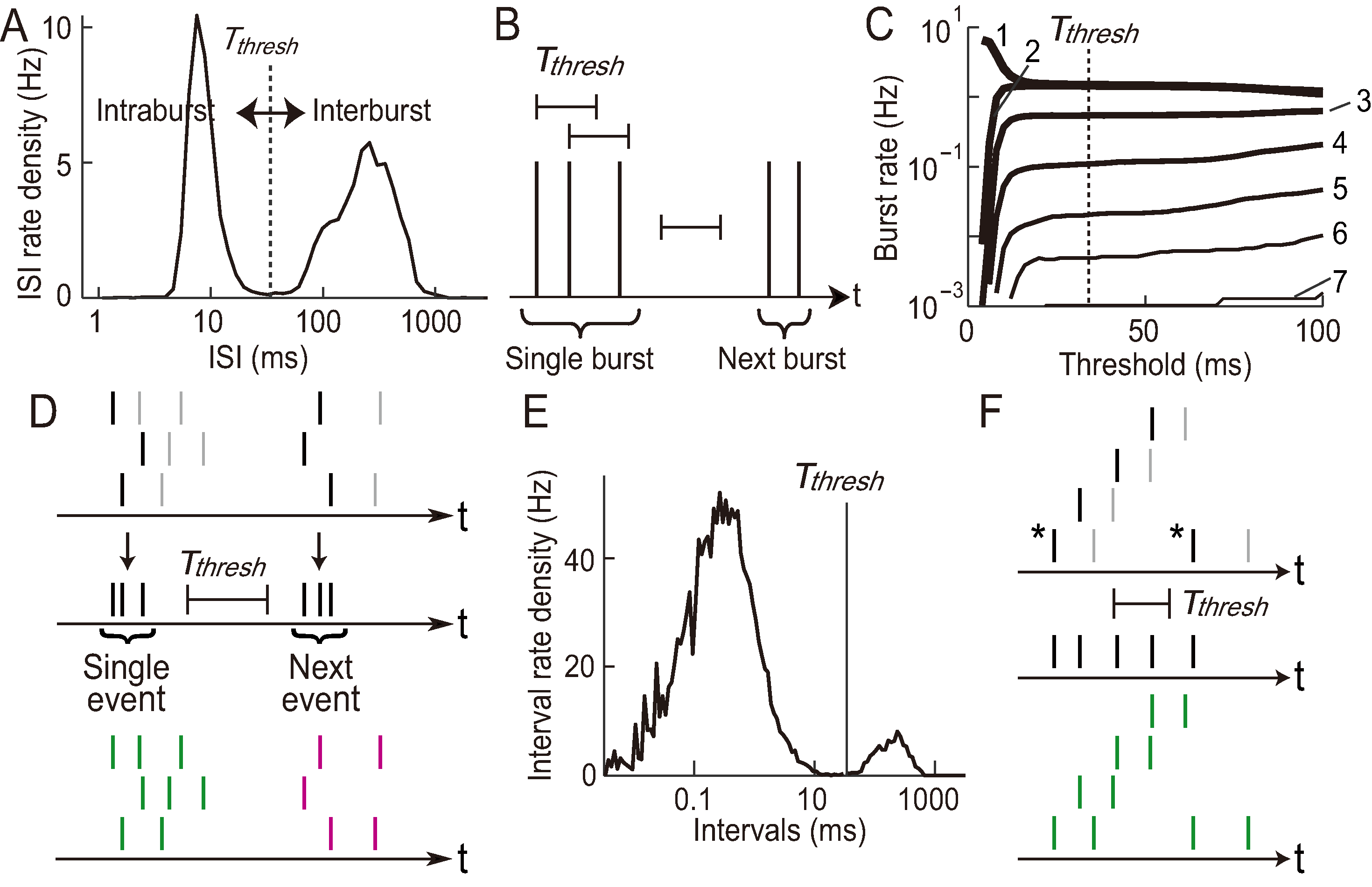

Supplement: S1 Fig — (A–C) Identification of bursts. (A) ISI histogram. Tthresh indicates the threshold interval, which was set at the trough between the two peaks in the histogram. (B) Algorithm used to define bursts. The vertical lines represent spikes. When two consecutive spikes occurred with an interval shorter than Tthresh, they were incorporated into the same burst. If the interval was longer than Tthresh, the two consecutive spikes were separated into two different bursts. (C) Rates of isolated spikes (1) and bursts with 2–7 spikes (2–7) plotted against the threshold interval. Data from the cell shown in Fig 1. (D–F) Identification of events. (D) Algorithm used to define events. The top panel shows a schematic raster plot. Each row shows spikes that occurred during a single repeat of the stimulation. The first spikes of the bursts (black lines at the top) were merged into a single train (middle). When the two first spikes in the merged train were closer than Tthresh, they were incorporated into the same event; otherwise, they were assigned into two different events (bottom). (E) The intervals of the merged train of first spikes were determined and their histogram is shown. Tthresh indicates the threshold interval. Data from the cell shown in Fig 1. (F) Exceptional case of the event identification. The top panel presents a schematic raster plot. Each row shows spikes that occurred during a single repeat of the stimulus. The black lines in the top panel represent the first spikes of bursts. The timing of the bursts shows a large jitter in different repeats. The asterisks indicate two consecutive bursts. The middle panel shows the merged train of the first spikes. Because all intervals were <Tthresh, all bursts were incorporated into the same event (bottom). The two consecutive bursts marked by the asterisks in the top panel were merged into one burst. See also Materials and Methods. (TIF) [file pcbi.1007726.s001.tif]

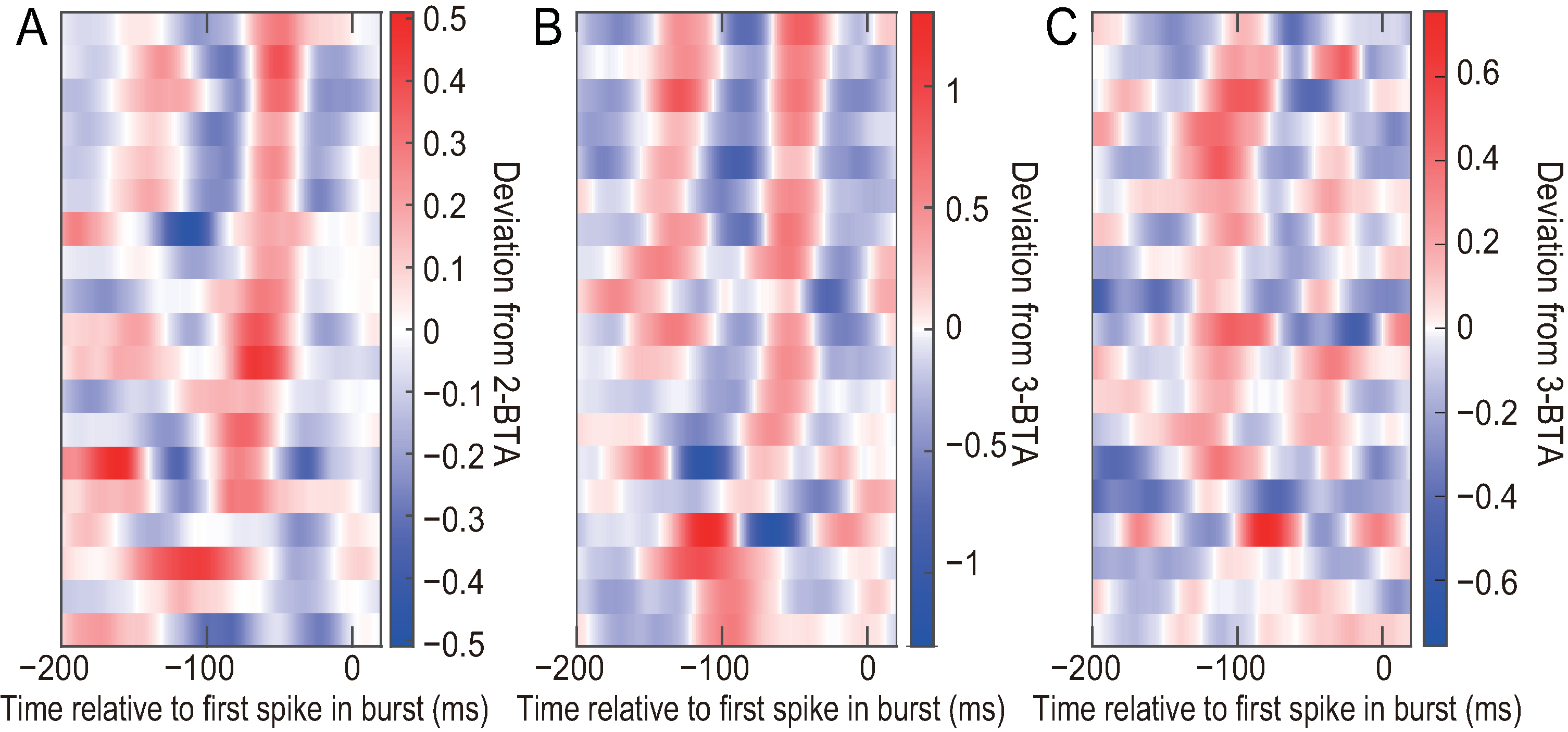

Supplement: S2 Fig — (A) Stimulus sequences encoded by 2-spike burst ISIs. Each row represents one of the 19 cells that generated at least 1500 2-spike bursts. For each cell, the deviation from 2-BTA was calculated by subtracting 2-BTA from the average of the stimulus sequence preceding the 2-spike bursts with the longest and shortest 50% of intraburst ISIs (see yellow and blue lines in Fig 3G, respectively). The difference in the deviation from 2-BTA for the longest and shortest ISIs are shown. (B and C) Stimulus sequences encoded by the two approximately independent components of 3-spike bursts. Data for the 19 cells that generated at least 1200 3-spike bursts. (B). The k-th row represents Dev1+,k(τ)−Dev1−,k(τ), i.e., the difference in the average stimulus sequence preceding 3-spike bursts with the burst phase within the range centered by θ and θ + 180°, where θ represents the angle between the u1 axis and the principle axis with the smaller variance (see Fig 8E). (C) The k-th row represents Dev2+,k(τ)−Dev2−,k(τ), i.e., the difference in the average stimulus sequence preceding 3-spike bursts with the burst phase within the range centered by θ + 90° and θ + 270°, calculated for the k-th cell. See Materials and Methods. (TIF) [file pcbi.1007726.s002.tif]

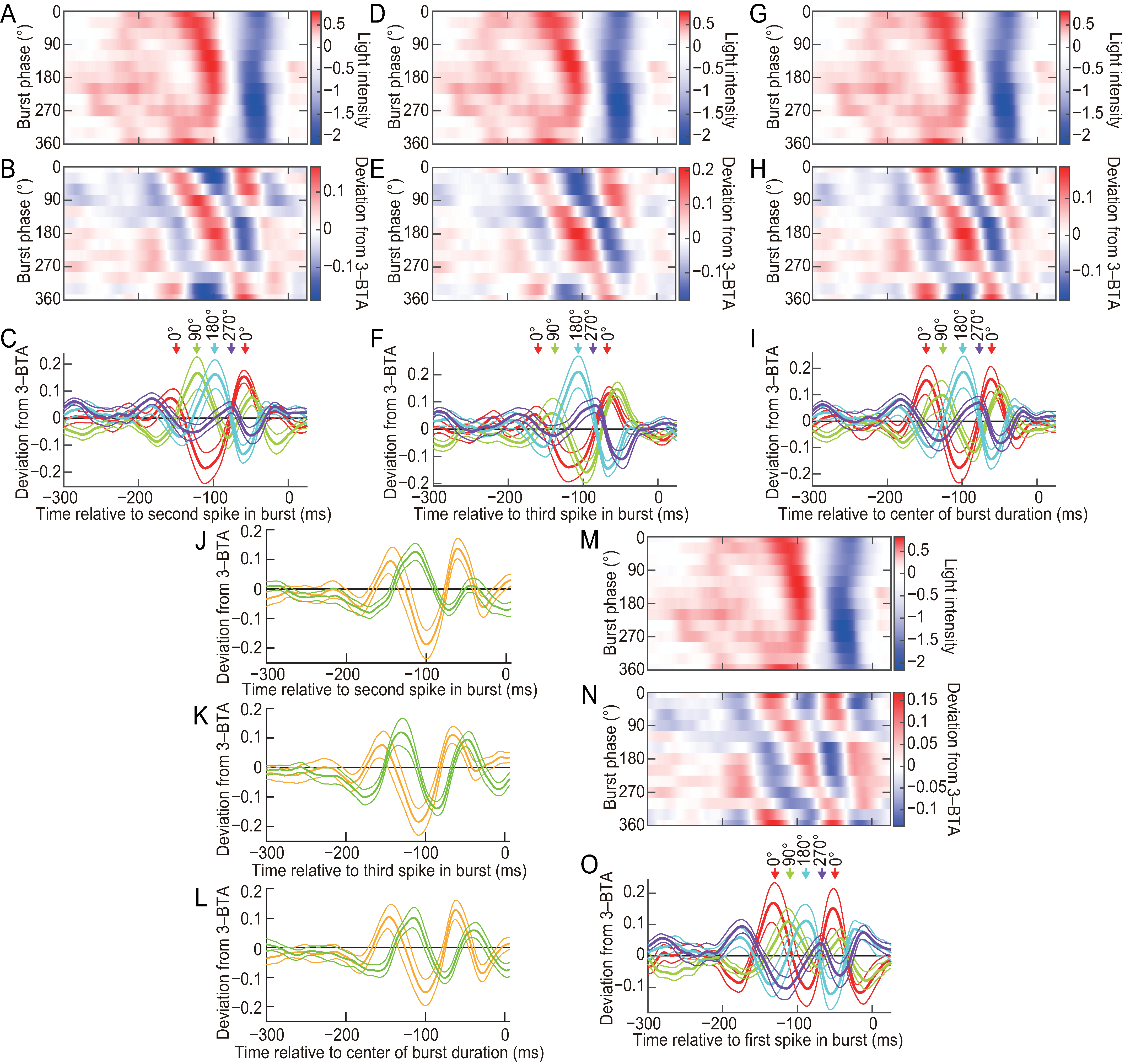

Supplement: S3 Fig — (A–L) Analysis of the effect of the stimulus alignment. (A–C) Stimulus sequences were aligned on the second spike in bursts. (A) Bursts were grouped according to the burst phase with the binwidth of 30°. Stimulus sequences preceding bursts in each group were averaged. Data from the cell shown in Fig 5A (compare with Fig 5A). (B) 3-BTA was calculated using preceding stimulus sequences aligned on the second spike in bursts. The deviation of 3-BTA was calculated by subtracting the 3-BTA from the data in (A). The panel shows the deviation of 3-BTA averaged across the 19 cells that generated at least 1200 3-spike bursts. Compare with Fig 5D. (C) The thick lines indicate the deviation of 3-BTA at the indicated burst phases averaged across the 19 cells. The thin lines are the SEM calculated across the cells. Compare with Fig 5E. (D–F) Analysis similar to that described in (A–C), with the exception that the stimulus sequences were aligned on the third spike in bursts. (G–I) Analysis similar to that described in (A–C), with the exception that the stimulus sequences were aligned on the middle of the duration of bursts, i.e., the middle between the first and third spikes. (J–L) Stimulus sequences encoded by the independent components w1 and w2. Stimulus sequences were aligned on the second spike (J), the third spike (K), and the middle of the duration of the bursts (L). For each cell, the deviations from 3-BTA were calculated by subtracting 3-BTA from the average stimulus sequence preceding 3-spike bursts with the burst phase within the range centered by θ (for w1) and θ + 90° (for w2), where θ represents the angle between the u1 axis and the principle axis with the smaller variance (see Fig 8E). The thick lines indicate the deviation from 3-BTA averaged across 19 cells that generated at least 1200 3-spike bursts (yellow: w1; green: w2). The thin lines show SEM values calculated across the cells. (M–O) Bursts excluded from the analysis because of small ISIs were incorporated i [file pcbi.1007726.s003.tif]

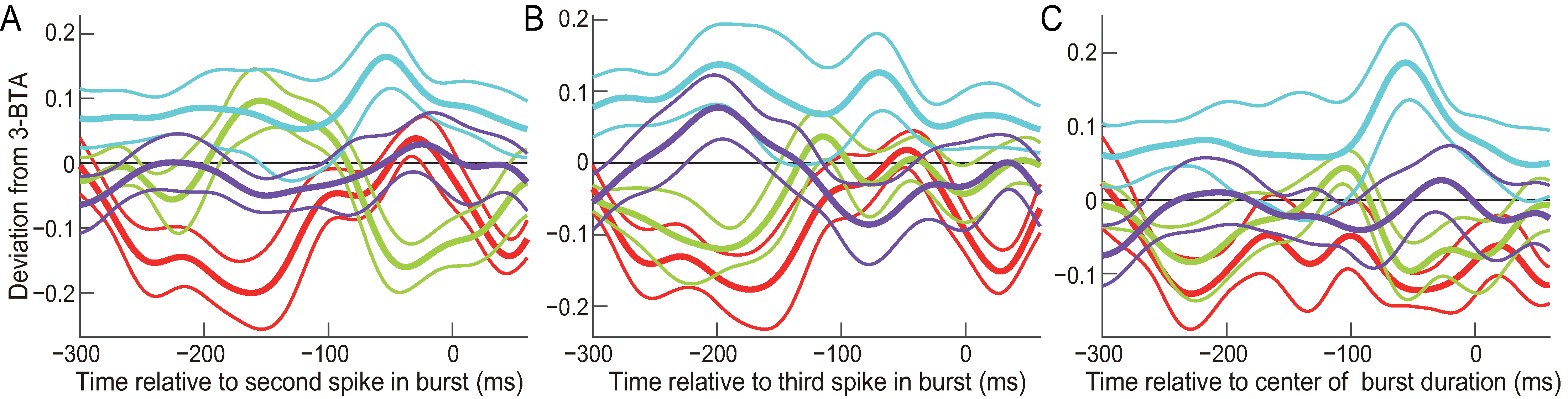

Supplement: S4 Fig — The deviation from 3-BTA was calculated by aligning bursts on the second spike (A), third spike (B), and middle of the duration (C) of bursts. For each cell, the deviation from 3-BTA was calculated by subtracting 3-BTA from the average stimulus sequence preceding 3-spike bursts with the burst phase within a range centered by 0° (red), 90° (green), 180° (cyan), and 270° (magenta). The average (thick lines) and SEM (thin lines) among the 8 cells that generated more than 1000 3-spike bursts are shown. Compare with Fig 6D. (TIF) [file pcbi.1007726.s004.tif]
