# Supplementary material for: Interspike intervals within retinal spike bursts combinatorially encode multiple stimulus features
Source: PLoS Comput Biol. 2020 Nov 6;16(11):e1007726. doi: 10.1371/journal.pcbi.1007726 (PMC7738174; doi:10.1371/journal.pcbi.1007726)
Supplement: Attachment — Submitted filename: review_plos_2020.pdf [file pcbi.1007726.s005.pdf]

1. Overall

The topic and claim are an interesting: a form of multiplexing in burst coding, where intraburst ISIs and the number of spikes in a burst code for independent features of the stimulus. This is interesting and novel, and the experimental paradigm is correct for this question. However, I find the explanation of the analysis a bit too minimal, which makes it hard for me to judge whether it is done correctly (see for more details below and comments in the text). I think it is, but I cannot always tell. Also, the provided data and code lacks a "readme" or other documentation/instructions, which makes it hard to use.

Secondly, the main claim of the paper is shown in figure 5-7. However, this depends critically on how bursts of different duration are aligned (first spike, last spike, middle spike, middle of the total duration). With the coordinate transformation, it is a bit lost how bursts are aligned exactly. Could the authors explain how these bursts are aligned, and argue why the results in figs. 5-7 are not simply an effect of burst alignment (or alternatively, why this alignment is indeed the relevant one for the next processing layer, the visual thalamus)?

Finally, the frequency of the components shown in figure 5 and 7 are exactly the temporal resolution of the stimulus (30 Hz). The components for the natural movie in figure 6 (C and D) are much slower (about 10 Hz). So do these 'deviations from the BTA'-components depend on the autocorrelation of the stimulus? What was the autocorrelation of the stimulus for the natural movies and is there a relation with the found components?

So my recommendation is that the authors should:

- Explain better how events were determined and why there is such a big difference between burst-threshold-ISI between the Gaussian sequence and the natural movie.
- Explain more the reasoning of their MI calculation: the MI between what and what is calculated, and why? Why is a single spike counted as a burst?
- Explain the BTA subtraction, and include a calculation of projection values (see for instance figure 1C of Slee, S. J., Higgs, M. H., Fairhall, A. L., & Spain, W. J. (2005). Two-Dimensional Time Coding in the Auditory Brainstem. The Journal of Neuroscience, 25(43), 9978–9988.) to substantiate their claims.
- Explain the reconstruction method in more detail than the two sentences currently in the methods.
- Expand the explanations in figure captions.
- Improve the mathematical notation. For instance, always say about posterior entropies, mutual information, etc between what and what it is calculated, and don't use the same names for both base vectors and the components of a vector (u1 and u2).
- Add documentation to the code/data

2. Details

2.1 Data/Code

I looked at the link (https://osf.io/29ect/), but there is no README file or something similar, so it is very difficult to get started with the data. Could the authors include some documentation about the data and code, and how to use it?

2.2 Figures

- Figure 1: the description in the figure caption is very minimal, especially for C and E: either move this to the corresponding section (methods), or give a bit more explanation.
- Figure 2, 3, 5:
    - How can the BTA be so smooth, when the temporal resolution of the stimulus was 30 Hz (i.e. about 33 ms per frame)? Was some sort of regularization used? If so, which? It is not described in the methods.
    - 2A, 3E, 5F, If you really want to know whether "the number of spikes within a burst encodes the amplitude of ON-to-OFF light intensity modulation", you need to normalize the BTAs and calculate the projection values of burst-triggered versus random-triggered events (see for instance figure 1C of Slee, S. J., Higgs, M. H., Fairhall, A. L., & Spain, W. J. (2005). Two-Dimensional Time Coding in the Auditory Brainstem. *The Journal of Neuroscience*, *25*(43), 9978–9988.)
    - 2B, 3F&G, 5D : did you subtract the means, or on a cell-by-cell basis? How was the STD calculated? Please give a description in the methods and refer to this in the text and figure caption!
- Figure 5
    - This is one of the most important claims of the paper: the fast that the BTA is phase-shifted depending on the ISIs. However, this claim depends critically on how bursts of different duration are aligned (first spike, last spike, middle spike, middle of the total duration). So could the authors explain how these bursts are aligned (i.e. how the coordinate transformation affect alignment), and argue why the results in fig. 5 E&G is not simply an effect of burst alignment?
    - G-I: the explanation in the caption is not enough to follow what is going on here. Please explain what is done and why. Also, these are not referred to in the main text, please add it to the general story.
- Figure 6
    - See figure 5: explain burst alignment.
    - B: the last 2 groups (6.4-14.5 ms and < 6.4 ms) have the same color in the pdf.
    - D: it is not explained what the arrows indicate.
- Figure 7:
    - C: where are the data points? Could these be added? Also, could the axis have ticks+numbers, so that B and C can be compared (I suppose the magenta line should be in the same direction)? Or alternatively, could C and D be reversed in order (then it makes a lot more sense)?
    - E-F: please, don't just say 'the same as some other figure', but explain, that makes it so much easier to read.

2.3 Main text

- Line 134: "Two-spike burst ISIs were not correlated to the average number of spikes in events (Fig 3B…" I would expect for this claim, a plot of the ISIs on one axis, and the number of spikes in an event on the other, with a correlation measure. However, here you just see for 4 events the ISIs. So this does not show whether there is a correlation between ISIs and number of spikes or not.
- Line 104, 147: I think for these claims, projection values need to be calculated (see for instance figure 1C of Slee, S. J., Higgs, M. H., Fairhall, A. L., & Spain, W. J. (2005). Two-Dimensional Time Coding in the Auditory Brainstem. The Journal of Neuroscience, 25(43), 9978–9988.)
- Line 180: Explain how this linear and non-linear scaling was done. There is some text in the methods, but it is not referred to, and also explain the gist of it in the main text.
- Line 183: you use u1 and u2 for both the base vectors and their components. This is unclear mathematical notation.
- Line 232 and further: I get lost here: you're comparing two groups with a separation of 80 ms, but both have phase 0, so what is it that you're comparing? After this: how was the reconstruction done exactly? I tried to infer it from the methods, but not enough detail was provided. What does "estimated stimulus sequence by adding the STA aligned according to the burst spikes" mean? Do you generate an STA for every type of burst? But what do you add then? Or do you generate an STA for a silence of length x + spike sequence?

2.4 Discussion

Good that both possible encoding and decoding mechanisms are discussed! Since you mention a variation of ISI sensitivity: Did you observe neural homogeneity or heterogeneity in your recorded neurons? Did you find all neurons code for exactly the same features? In the case of 2-spike bursts, did they encode the same features at the same phase? Or was there variability between the neurons?

2.5 Methods

Burst and event definition

- There is quite a bit difference between the threshold-ISI for bursts for the Gaussian sequence and the natural movie. Could the authors please share their thoughts on
- I don't understand the event extraction, the description is not quite clear (although I do understand the gist of it). Could it please be clarified?

Mutual information of spike number in a burst:

- The mutual information calculation is a bit strange: the authors seem to calculate the mutual information between the number of spikes in a burst (k) and the event number j. However, they never state this explicitly. Moreover, why is this a relevant mutual information to calculate? I would say that the receiver, i.e. the visual thalamus, cares about the stimulus, not about the event number? Maybe there is a good reason for calculating this, but the authors don't explain this.
- A single spike is counted as a burst??

Mutual information of ISIs:

- The fact that you divide the ISIs in four groups of equal size, means that you exclude the option of ‘rare but very informative events’ occurring. Could the authors explain why they made this decision?

Coordinate transformations

- I understand the gist of what you do, but again, some information is lacking.
- Why were events with small ISI excluded? Doesn’t this bias the results?
