# Supplementary material for: Interspike intervals within retinal spike bursts combinatorially encode multiple stimulus features
Source: PLoS Comput Biol. 2020 Nov 6;16(11):e1007726. doi: 10.1371/journal.pcbi.1007726 (PMC7738174; doi:10.1371/journal.pcbi.1007726)
Supplement: Attachment — Submitted filename: RevisionLetter200711_2346 _bind.pdf [file pcbi.1007726.s007.pdf]

Response to Reviewers

Response to Reviewer 1

Reviewer #1: In their manuscript "Interspike intervals within retinal spike bursts combinatorially encode multiple stimulus features" the authors analyze the relation between intraburst ISIs of 2-spike or 3-spike bursts and stimulus features on data recorded from salamander retinal ganglion cells. The authors identify stimulus features encoded by the different ISIs. The analysis is timely and very naturally extends previous studies which showed that the number of spikes within a burst, the burst onset and duration of a burst carry stimulus information. The manuscript is very well written and the figures are clear and informative.

Major point: There is a conceptual issue with the mutual information analysis. A couple of times (lines 98, 116, 152), the authors mention the "information about the stimulus". Intuitively, I would expect that stimulus here is referring to the light intensity profile. But from Materials and Methods, it becomes clear that the authors are referring to information about "event" identity. The authors define "events" as reproducible bursts. Essentially, an event is not an external stimulus variable but itself a neural response property which happens to be linked to the external stimulus onset. However, even for constant stimuli, RGCs can elicit complex patterns for hundreds of milliseconds similar to the patterns shown in Fig 1. Thus, there seems to be a conflation of the firing dynamics of the RGCs and the light intensity profile in the mutual information analysis. Therefore, it seems the authors show that they can distinguish between different time points relative to a stimulus onset but not necessarily between different stimuli i.e. between different intensity profiles or between different intensities within a profile. In my opinion, this is not "information about the stimulus". This concerns the mutual information analysis only. The authors also evaluate relations of intraburst ISIs to amplitudes and temporal phases of oscillatory components which I find convincing.

Response: We thank the reviewer for raising this issue regarding the information calculations. We agree that the original information calculation method used in the manuscript had issues. The revised manuscript uses a different method that provides a better estimation. As indicated by the reviewer, our original method calculated the mutual information between burst ISI patterns and the event identity. This method relied on an implicit assumption that information about the event identity is similar to information about the stimulus. However, as the reviewer suggests, if the spike number and/or ISIs of consecutive events are not independent (e.g., thorough intrinsic dynamics of the RGCs), the information concerning the event identity can have a complicated

relationship with the information concerning the stimulus.

Therefore, the revised manuscript utilizes a method that does not rely on hypotheses about encoding and provides a better estimation of information about the stimulus (Brenner et al., Synergy in a Neural Code, Neural Computation 12, 1531, 2000). The revised information values tended to be larger than those calculated in the original analysis and supported our previous conclusions.

We would again like to thank the reviewer for giving us the opportunity to correct the previous analysis.

Minor points:

The authors analyzed 2-spike and 3-spike bursts only and state that bursts with 4 or more spikes were too rare to conduct a similar analysis (line 314 and Fig 1D, though the illustrated green reproducible burst in Fig 1A then appears to be unusual). I was wondering, could anything be said when pooling together all bursts with more than 3 spikes? Do the first two intraburst ISIs hint at the same characteristics as for the 3-spike bursts? An answer to this question would shed light on the general validity of the amplitude and phase encoding.

Response: We thank the reviewer for this interesting suggestion. To investigate this issue, we extracted the first and second ISIs of bursts with 4 or more spikes. We then calculated the mutual information about the stimulus conveyed by these ISIs. Interestingly, the mutual information was as large as that conveyed by 3-spike bursts, suggesting that the first and second ISIs convey information regarding visual input. We also intended to analyze the triggered averages, but it was difficult to obtain conclusive results, likely because bursts with 4 or more spikes were relatively rare. We describe this issue in the discussion.

Line 480: typo: insert "in" before "the open"

Response: We thank the reviewer for making us aware of this typo. It has been corrected.

We would like to notify the reviewer of several changes in the revision, which do not affect the conclusions. First, the original Fig 5A showed more than 2,800 stimulus sequences that each preceded a burst. In order to show these sequences, we had smoothed the color-coded stimulus sequences in the vertical orientation using sliding-window averaging with a window width of 60°. We apologize for not describing this process in the original manuscript. Because the 60° window width was a bit too large and simple averaging is easier to interpret, we changed the reporting method to simple binning and averaging, as reported in “Deviation from 3-BTA” in the revised Materials and Methods section. Three-spike bursts were divided into 12 groups according to the

burst phase. The lower and upper phase limits for the bins were $0° \pm 15°, 30° \pm 15°, \cdots, 330° \pm 15°$. The preceding stimulus sequences were collected and averaged for each of the 12 burst groups. The 12 average sequences are now shown in the revised Fig 5A. Fig 5C, 5D, and 5H were similarly revised, with 5E and 5I revised accordingly. The results in the revised figures are very similar to what we originally reported, indicating that the revision had little to no effect on the results.

Second, in the coordinate transformation of 3-spike burst ISIs, variables are normalized before the principle component analysis, but we failed to explain this normalization in the original manuscript. In addition, we found that the normalization used in original Figs 6D and 7 was different from that for Fig 5. In the revised manuscript we have described the normalization, removed the principle axes in Fig 5D, and performed the analysis in Figs 6D and 7 using the same normalization that is used in Fig 5. Revised Fig 6 presenting the natural scene experiment shows that the burst phase encodes the temporal phase of the deviation from 3-BTA like the original manuscript. However, the correlation appears to be weaker than that in the original analysis (revised Fig 6D). We consider this issue in the discussion section ("Generality of the combinatorial ISI coding"). Revised Fig 7 is very similar to original Fig 7. The orientation of the steepest gradient in Fig 7B (original: 33.1° ± 15.6°, revised: 32.3° ± 15.6°; Fig 7D, magenta) and the orientation of the principle axes in Fig 7C (original: 29.4° ± 7.3° and 119.4° ± 7.3°, revised: 28.6° ± 7.7° and 118.6° ± 7.7°, respectively; Fig 7D, yellow and green, respectively) have been revised accordingly in the main text. We apologize for the initial issue with the coordinate transformation.

Third, in Figs 4B, 4D, 4E, 7C, 7E, and 7G, ~1.5% of data points are out of the range of the plot. We now describe this issue in the figure captions.

Response to Reviewer 2

Reviewer #2: This is a very interesting study supporting the multiplexed encoding of stimulus information in a combinatorial burst code. The authors analysed data from retinal ganglion cells of salamander larvae when stimulated with oscillatory light stimuli and natural scenes. The main result is that intra-burst ISIs encode faster features of the stimuli in comparison with spike number alone. Moreover, the study shows that ISIs can combinatorially encode different features of the stimulus. The analyses are done with great attention to detail and there is an overall excellent quality of presentation.

Response: We thank the reviewer for their positive response to the manuscript. We believe that

the revisions made in response to the other reviewers have further improved the paper.

We would like to notify the reviewer of several changes in the revision, which do not affect the conclusions. First, the original Fig 5A showed more than 2,800 stimulus sequences that each preceded a burst. In order to show these sequences, we had smoothed the color-coded stimulus sequences in the vertical orientation using sliding-window averaging with a window width of 60°. We apologize for not describing this process in the original manuscript. Because the 60° window width was a bit too large and simple averaging is easier to interpret, we changed the reporting method to simple binning and averaging, as reported in “Deviation from 3-BTA” in the revised Materials and Methods section. Three-spike bursts were divided into 12 groups according to the burst phase. The lower and upper phase limits for the bins were $0^\circ \pm 15^\circ, 30^\circ \pm 15^\circ, \cdots, 330^\circ \pm 15^\circ$. The preceding stimulus sequences were collected and averaged for each of the 12 burst groups. The 12 average sequences are now shown in the revised Fig 5A. Fig 5C, 5D, and 5H were similarly revised, with 5E and 5I revised accordingly. The results in the revised figures are very similar to what we originally reported, indicating that the revision had little to no effect on the results.

Second, in the coordinate transformation of 3-spike burst ISIs, variables are normalized before the principle component analysis, but we failed to explain this normalization in the original manuscript. In addition, we found that the normalization used in Figs 6D and 7 was different from that for Fig 5. In the revised manuscript we have described the normalization, removed the principle axes in Fig 5D, and performed the analysis in Figs 6D and 7 using the same normalization that is used in Fig 5. Revised Fig 6 presenting the natural scene experiment shows that the burst phase encodes the temporal phase of the deviation from 3-BTA like the original manuscript. However, the correlation appears to be weaker than that in the original analysis (revised Fig 6D). We consider this issue in the discussion section ("Generality of the combinatorial ISI coding"). Revised Fig 7 is very similar to original Fig 7. The orientation of the steepest gradient in Fig 7B (original: 33.1° ± 15.6°, revised: 32.3° ± 15.6°; Fig 7D, magenta) and the orientation of the principle axes in Fig 7C (original: 29.4° ± 7.3° and 119.4° ± 7.3°, revised: 28.6° ± 7.7° and 118.6° ± 7.7°, respectively; Fig 7D, yellow and green, respectively) have been revised accordingly in the main text. We apologize for the initial issue with the coordinate transformation.

Third, in Figs 4B, 4D, 4E, 7C, 7E, and 7G, ~1.5% of data points are out of the range of the plot. We now describe this issue in the figure captions.

Response to Reviewer 3

1. Overall

The topic and claim are an interesting: a form of multiplexing in burst coding, where intraburst ISIs and the number of spikes in a burst code for independent features of the stimulus. This is interesting and novel, and the experimental paradigm is correct for this question. However, I find the explanation of the analysis a bit too minimal, which makes it hard for me to judge whether it is done correctly (see for more details below and comments in the text). I think it is, but I cannot always tell.

Response: We apologize for the insufficient analysis description in the original manuscript. We have thoroughly revised the description in the results and methods to clarify our explanation of the analyses.

Also, the provided data and code lacks a "readme" or other documentation/instructions, which makes it hard to use.

Response: We apologize for the insufficient data and code information. We have provided the documentation and instructions in the revision.

Secondly, the main claim of the paper is shown in figure 5-7. However, this depends critically on how bursts of different duration are aligned (first spike, last spike, middle spike, middle of the total duration). With the coordinate transformation, it is a bit lost how bursts are aligned exactly. Could the authors explain how these bursts are aligned, and argue why the results in figs. 5-7 are not simply an effect of burst alignment (or alternatively, why this alignment is indeed the relevant one for the next processing layer, the visual thalamus)?

[*Could the authors explain how these bursts are aligned*]

Response: We apologize for the insufficient explanation. The bursts are aligned with the first spikes. Thus, in Fig 5 (except panel 5F), "0" on the horizontal axes indicates the time of the first spike in each burst. Therefore, the time measurements on the horizontal axes are relative to the first spike (i.e., the burst onset). The vertical axes in Fig 5A, 5C, 5D, and 5H of the revised manuscript correspond to the burst phase defined in Fig 4E. Therefore, the coordinate transformation affected the order within the vertical axes, but not the alignment in the horizontal axes. The revised Materials and Methods includes a mathematical expression of the alignment in the "Burst-triggered average (BTA)," "Deviation from 2-BTA," "Deviation from 3-BTA," and

"Approximately independent components" sections. In addition, we changed the horizontal axes labels from “Time relative to burst onset” to “Time relative to first spike in burst.”

[*and argue why the results in figs. 5-7 are not simply an effect of burst alignment* (*or alternatively, why this alignment is indeed the relevant one for the next processing layer, the visual thalamus*)]

Response: To evaluate the potential effect of the burst alignment, we conduct the same analysis that was used in Fig 5, except that bursts are aligned with the second spike, third spike, and middle of the total duration (S3 Fig). The results (S3A–S3I Fig) are similar to those found when aligning with the first spike (Fig 5A, 5D, and 5E). The stimulus sequences preceding the bursts (S3A, S3D, and S3G Fig) are dependent on the burst phase, similar to the pattern shown in Fig 5A. The deviation from 3-BTA also shows a similar burst phase dependence (comparingS3B, S3E, and S3H Fig to Fig 5D). Although the deviation sequences are slightly distorted (comparing S3C, S3F, and S3I Fig to Fig 5E), they are all oscillatory sequences. Finally, their peaks (S3C, S3F, and S3I Fig) have temporal shifts like those shown in Fig 5E. Thus, aligning the burst to the first, second, or third spikes or the middle of the total duration produce similar results for the analysis shown in Fig 5. We discuss this issue in the last paragraph of "The two ISIs of three-spike bursts encode the phase and amplitude of oscillatory components" in Results.

We also investigated the effect of the alignment for bursts elicited by the natural scene movie (Fig 6 and S4 Fig). The natural movie-driven bursts showed greater dependence on the stimulus alignment (comparing S3A–S3I to S4 Fig), presumably because of the longer burst duration (~15.4 ms for the spatially uniform stimulus and ~29.0 ms for the natural scene movie). Aligning with the first spike in the bursts resulted in a similar phase dependence for the spatially uniform stimulus and natural scene movie (Fig 5 and Fig 6), indicating that the brain may use the first spike as its temporal reference to interpret the stimulus. We discuss this issue in the last paragraph of "The two ISIs of three-spike bursts encode the phase and amplitude of oscillatory components" in the Results and in "Generality of the combinatorial ISI coding" in the Discussion.

Finally, we analyzed the potential effect of the alignment on the analyses shown in Fig 7. Fig 7A–7E explain the relation between 2-spike burst ISI and 3-spike burst patterns and describe the coordinate transformation. Thus, these panels are irrelevant to burst alignment. We investigated whether the stimulus sequences encoded by the two independent components shown in Fig 7F depend on the alignment. S3J–S3L Fig show the stimulus sequences encoded by the two independent components, calculated for different alignments. Although the sequences show

some distortion, all of them are oscillatory sequences, and the two components have a phase shift of ~$1/4$ cycles. Thus, aligning the burst to the first, second, or third spikes or the middle of the total duration does not affect the results in Fig 7. This analysis is mentioned in the end of the third paragraph of "Two independent components of the burst patterns" section in the Results.

In sum, these results suggest that the data presented in Figs 5–7 are not simply an effect of burst alignment. We now consider this issue in the discussion section titled "Generality of the combinatorial ISI coding."

Finally, the frequency of the components shown in figure 5 and 7 are exactly the temporal resolution of the stimulus (30 Hz). The components for the natural movie in figure 6 (C and D) are much slower (about 10 Hz). So do these 'deviations from the BTA'-components depend on the autocorrelation of the stimulus? What was the autocorrelation of the stimulus for the natural movies and is there a relation with the found components?

Response: The temporal constant $\tau_{1/2}$, which represents the half-width at half height of the autocorrelation, was ~90 ms for the natural movie and ~33 ms for the spatially uniform stimulus. Therefore, the deviation from the BTA appears to depend on the autocorrelation of the stimulus. This suggests that the speed of the stimulus and the encoded components are related. We discussed this issue in the "Generality of the combinatorial ISI coding" section in the discussion.

So my recommendation is that the authors should:

Explain better how events were determined and why there is such a big difference between burst-threshold-ISI between the Gaussian sequence and the natural movie.

[Explain better how events were determined]

Response: We apologize for the insufficient description in the method. We have revised the event determination text ("Identification of Bursts and Events" section in revised Materials and Methods) and figure (S1D–S1F Fig).

[*why there is such a big difference between burst-threshold-ISI between the Gaussian sequence and the natural movie*]

Response: As indicated by the reviewer, the threshold-ISIs for the Gaussian sequence and the natural movie stimuli were ~40 ms and ~90 ms, respectively. Our method did not manually select these thresholds but set them at the lowest point of the trough between the two peaks observed in the ISI histogram. Therefore, the threshold-ISIs were set at ~40 ms and ~90 ms

because the spike firing is correlated up to ~40 ms and ~90 ms for the Gaussian and natural movie stimuli, respectively.

On the other hand, the temporal constant $\tau_{1/2}$, which represents the half-width at half maximum of the autocorrelation, was 33 ms and 90 ms for the Gaussian and natural movie stimuli, respectively (please see the revised Materials and Method). These values indicate that the light intensity value for each stimulus is similar for ~33 ms and ~90 ms. Therefore, spike firing elicited by the stimuli is likely correlated for ~33 ms and ~90 ms for the Gaussian and natural movie stimuli, respectively. These differences in the firing correlation would cause differences in the ISI histogram, leading to differences in the burst-threshold-ISIs. Therefore, the difference in the burst-threshold-ISIs likely reflects the difference in the temporal correlation of the stimuli.

We would like to note that although the threshold-ISI is ~90 ms for the natural movie, the median burst duration is much shorter (2-spike bursts: 15.4 ± 5.6 ms; 3-spike bursts: 29.0 ± 9.8 ms).

Explain more the reasoning of their MI calculation: the MI between what and what is calculated, and why? Why is a single spike counted as a burst?

[*the MI between what and what is calculated, and why?*]

Response: We thank the reviewer for raising the issue of information calculation. In the original manuscript, we calculated the mutual information between the number of spikes and the event identity and between the burst ISIs and the event identity. This method implicitly assumed that information about the event identity was similar to information about the stimulus. However, this assumption is not theoretically guaranteed. Indeed, information values calculated in this manner could have a complicated relationship with the stimulus information. First, repeating the stimulus causes bursts in the same event to occur at a slightly different time relative to the stimulus. This timing jitter affects the amount of information that can be obtained regarding the stimulus when the event identity is reported to the receiver. Moreover, different events can have different jitters that have varied effects on the information about the stimulus. Second, if the spike number and/or ISIs of consecutive events are not independent, the event identity information can have a complicated relationship with information about the stimulus.

Therefore, the revised manuscript utilizes a method that does not rely on hypotheses about encoding and provides a better estimation of information about the stimulus (Brenner et al., Synergy in a Neural Code, Neural Computation 12, 1531, 2000; "Mutual information" in revised Materials and Methods). The revised information values tended to be larger than those calculated

in the original analysis and supported our previous conclusions. We apologize for our original inaccurate calculation.

[*Why is a single spike counted as a burst?*]

Response: We apologize for our insufficient description. Ganglion cells often generate single spikes that are preceded and followed by longer intervals than T_{thresh}. We designate these as "isolated spikes." Isolated spikes can occur in events that have bursts with more than one spike (e.g., revised Fig 1, purple). Therefore, isolated spikes are effectively similar to bursts with more than one spike, and were thus treated as "1-spike" bursts. This definition is a natural generalization of bursts and enables us to use the same method for isolated spikes and bursts with ≥ 2 spikes (e.g., Fig 2).

Explain the BTA subtraction, and include a calculation of projection values (see for instance figure 1C of Slee, S. J., Higgs, M. H., Fairhall, A. L., & Spain, W. J. (2005). Two-Dimensional Time Coding in the Auditory Brainstem. The Journal of Neuroscience, 25(43), 9978–9988.) to substantiate their claims.

[*Explain the BTA subtraction*]

Response: We apologize for our insufficient description of these calculations. All the subtractions were done on a cell-by-cell basis. We now describe the detailed calculations in the revised "Burst-triggered average (BTA)," "Deviation from 2-BTA," "Deviation from 3-BTA," and "Approximately independent components" sections of the Materials and Methods and in the figure legends. These calculations are detailed below.

<u>"Burst-triggered average (BTA)" in the revised Materials and Methods</u>

In Fig 2B, the difference between the 3-BTA and 1-BTA (3-BTA–1-BTA) was calculated on a cell-by-cell basis. The mean ± SEM of 3-BTA–1-BTA was calculated across the 41 cells at each time point from −300 ms to 0 ms.

<u>"Deviation from 2-BTA" in the revised Materials and Methods</u>

Revised Fig 3F and 3G (Fig 3E and 3F in the original manuscript) show data from a single cell. The 2-BTA was first calculated for the cell (black in revised Fig 3F and original Fig 3E). Next, the 2-spike bursts of the cell with the longest ISIs (top 50%) were collected and the stimulus sequences preceding these bursts were determined. The 2-BTA of the cell was subtracted from these sequences, and the mean ± SEM were calculated across the sequences and are shown as

thick and thin yellow lines in revised Fig 3G (original Fig 3F). The stimulus sequences preceding the 2-spike bursts with the shortest ISIs (bottom 50%) were also processed and shown as the thick and thin blue lines in revised Fig 3G (original Fig 3F). These sequences shown as thick yellow and blue lines in revised Fig 3G (original Fig 3F; termed the deviations from 2-BTA) were similarly calculated for all 41 cells. The mean ± SEM of these 41 sequences were calculated and shown in the thick and thin lines in the revised Fig 3H (original Fig 3G).

Original Figure 5

Original Fig 5 was generated using the following steps. First, for each cell, the burst phase was defined for each 3-spike burst (Fig 4E). For each phase $\theta = 0°, 1°, 2°, \cdots, 359°$, bursts with a phase larger than $\theta - 30°$ and smaller that $\theta + 30°$ were identified, and the stimulus sequences preceding these bursts were averaged. Original Fig 5A shows these 360 sequences calculated for the cell used in Fig 1 (“0” on the horizontal axis in original Fig 5A indicates the time of the first spike in the burst). The 3-BTA was also calculated for each cell (original Fig 5B). The 3-BTA was subtracted from the 360 sequences in original Fig 5A, and the resultant 360 sequences were shown in original Fig 5C. The 360 sequences similar to Fig 5C were obtained for all cells and averaged across cells to generate original Fig 5D.

Revised Fig 5 and “Deviation from 3-BTA” in the revised Materials and Methods

Because the 60° window width was a bit too large and simple averaging is easier to interpret, we changed the reporting method to simple binning and averaging, as reported in “Deviation from 3-BTA” in the revised Materials and Methods section. For each cell, three-spike bursts are divided into 12 groups according to the burst phase. The lower and upper phase limits for the bins are $0° \pm 15°, 30° \pm 15°, \cdots, 330° \pm 15°$. The preceding stimulus sequences are collected and averaged for each of the 12 burst groups. The 12 average sequences are now shown in the revised Fig 5A. The 3-BTA is also calculated for each cell (revised Fig 5B). The 3-BTA is subtracted from the 12 sequences in revised Fig 5A, and the resultant 12 sequences are shown in revised Fig 5C. The 12 sequences corresponding to Fig 5C are obtained for all cells and averaged across cells to generate revised Fig 5D. Fig 5H is similarly revised, with 5E and 5I revised accordingly. The results in the revised figures are very similar to what we originally reported, indicating that the revision had little to no effect on the results.

Revised Fig. 7 and "Approximately independent components"

The stimulus sequences encoded by the two independent components shown in Fig 7F are calculated similarly to the sequences in Fig 5E except that they were calculated for the burst

phase range of $\theta \pm 15°$ and $(\theta + 90°) \pm 15°$, where θ represents the angle between the u_1 axis and the principle axis with the smaller variance (Fig 7C–7E).

[include a calculation of projection values to substantiate their claims]

Response: We appreciate the reviewer' suggestion to analyze the projection values. The revised manuscript calculates the stimulus values projected onto the BTAs. In accordance with Slee *et al.*, 2005, we normalized the prior distribution of the projection values (please see below). This normalization effectively generates the same results as the BTA normalization.

<u>Spike number (Revised Fig 2C, 2D and "Projection onto 3-BTA − 1-BTA" in the revised Materials and Methods).</u>

To analyze information coding by the spike number, we projected the stimulus onto 3-BTA−1-BTA of each cell. Revised Fig 2C shows the probability density distribution of the projection values for all stimuli (dashed line) and those triggered by isolated spikes, 2-spike bursts, 3-spike bursts, and 4-spike bursts (solid lines). The projection values triggered by bursts with a different number of spikes had different probability distributions. Moreover, this shift was larger for bursts with more spikes. These results indicate that the spike number conveys information about the stimulus feature represented by 3-BTA−1-BTA.

The above analysis compares projection values at randomly sampled time points and those sampled at bursts with k-spikes. Thus, the difference in the probability distribution represents two pieces of information: the information conveyed by a burst occurrence (including isolated spikes) and the information conveyed by the spike number. To visually conceptualize the latter, we collected the projection values at the time point of all bursts and isolated spikes and normalized them so that the distribution had a mean = 0 and a SD = 1. (Fig 2D, dashed line). The values for bursts with a specific number of spikes show different distributions than the values combined for all bursts and isolated spikes (Fig 2D). This result also demonstrates that the probability distribution of the projection values changes according to the spike number (Fig 2D), confirming that the spike number conveys information about the stimulus feature represented by the 3-BTA − 1-BTA.

<u>Two-spike burst ISI (Revised Fig 3I, 3J and "Deviation from 2-BTA" in the revised Materials and Methods)</u>

We also conducted projection analysis for 2-spike burst ISIs. The stimulus feature was the difference between the oscillatory features shown by the yellow and blue lines in revised Fig 3G

calculated for each cell. We collected the projection values at the time points of all 2-spike bursts and normalized the values. We then identified bursts with the shortest (0%–10%), intermediate (45%–55%), and longest (90%–100%) ISIs and collected the projection values at the time points of those bursts. The probability density distribution was determined for these values and the mean ± SEM calculated for the 41 cells is shown in Fig 3I. The probability distributions for the shortest and longest ISIs were shifted from the distribution for all 2-spike bursts combined and the shifts were in the opposite directions. This indicates that the ISIs convey information about the stimulus feature represented by the BTA.

We next conducted the same analysis but this time projecting the stimulus onto the 3-BTA − 1-BTA, which is the stimulus feature encoded by the spike number. Interestingly, 2-spike bursts with the shortest, intermediate, and longest ISIs all had projection value distributions that were almost indistinguishable from the distribution for all 2-spike bursts combined (revised Fig 3J). This result suggests that 2-spike burst ISIs convey little to no information about the stimulus feature encoded by the spike number. This result further suggests that 2-spike burst ISIs and the spike number encode different features about the stimulus. Therefore, the result is consistent with the data showing that 2-spike burst ISIs are not correlated with the average spike number in each event.

<u>Three-spike burst patterns (Revised Fig 7H–7K and "Approximately independent components" in the revised Materials and Methods)</u>

Finally, we performed projection analysis for 3-spike burst patterns. The oscillatory stimulus features $f_1(t)$ and $f_2(t)$ corresponding to the two approximately independent components w_1 and w_2 were determined as explained in the Materials and Methods.

In Fig 7H, $f_1(t)$ was used. The probability density distribution was calculated for all 3-spike bursts and for 3-spike bursts with small ($w_1 < -2$), intermediate ($-0.75 < w_1 < 0.75$), and large ($2 < w_1$) w_1 values (note that w_1 has a mean ~ 0 and SD ~ 1). The projection values collected for 3-spike bursts with small and large w_1 had probability distributions that were shifted to the left and right of that calculated for all 3-spike bursts, respectively (Fig 7H). Thus, the coordinate w_1 conveys information regarding the stimulus features represented by $f_1(t)$. We obtained similar results from the analyses for w_2 and $f_2(t)$ (Fig 7K), indicating that the coordinate w_2 conveys information regarding the stimulus features represented by $f_2(t)$. The shifts in the probability density distributions were larger for w_1 (Fig 7H and 7K) which is consistent with the analysis showing that w_1 is more informative than w_2.

To analyze the specificity of information coding, we projected the stimulus onto $f_2(t)$ and collected the value at 3-spike bursts with small, intermediate, and large w_1. Interestingly, the projection values triggered by different w_1 values had probability density distributions that were indistinguishable from all 3-spike bursts combined (Fig 7I). This result suggests that the w_1 value conveys little to no information about the stimulus feature represented by $f_2(t)$. Similar results were obtained for the analyses using w_2 and $f_1(t)$ (Fig 7J). Thus, w_1 and w_2 specifically convey information about the stimulus features represented by $f_1(t)$ and $f_2(t)$, respectively. This is consistent with the idea that w_1 and w_2 encode independent information.

In sum, the BTA projection analyses indicate that (1) the spike number within a burst encodes the stimulus feature represented by the 3-BTA − 1-BTA. (2) The ISI of 2-spike bursts encodes an oscillatory stimulus feature. (3) The burst ISI and spike number convey different information about the stimulus. (4) The two independent components of the 3-spike burst patterns encode two oscillatory stimulus features in a largely independent manner.

Thus, investigating the stimulus projections strengthened our original conclusions. We would like to thank the reviewer again for proposing the analyses.

Explain the reconstruction method in more detail than the two sentences currently in the methods.

Response: We apologize for the poor description of the reconstruction. The revised manuscript uses mathematical descriptions to detail our method ("Linear reconstruction" in the revised Materials and Methods).

• Expand the explanations in figure captions.

Response: We apologize for the insufficient figure captions. We have revised the captions by adding new sentences that provide further detail and clarity.

• Improve the mathematical notation. For instance, always say about posterior entropies, mutual information, etc between what and what it is calculated, and don't use the same names for both base vectors and the components of a vector (u1 and u2).

Response: We thank the reviewer for this suggestion to improve the mathematical notation. We have revised the mathematical notations including those for mutual information, coordinate transformation, and the use of u_1 and u_2.

• Add documentation to the code/data

Response: We apologize for not having provided sufficient information. The revised manuscript provides documentation to the data and code.

2. Details

2.1 Data/Code

I looked at the link (https://osf.io/29ect/), but there is no README file or something similar, so it is very difficult to get started with the data. Could the authors include some documentation about the data and code, and how to use it?

Response: We apologize for not having provided sufficient information for how to use the data and code. We now provide their documentation and explain how to use them.

2.2 Figures

• Figure 1:

o the description in the figure caption is very minimal, especially for C and E: either move this to the corresponding section (methods), or give a bit more explanation.

Response: We apologize for the insufficient caption. We have improved the explanation in the caption for original Fig 1B–1F and moved the panels to S1 Fig, and revised the method ("Identification of bursts and events" section in revised Materials and Methods).

• Figure 2, 3, 5:

o How can the BTA be so smooth, when the temporal resolution of the stimulus was 30 Hz (i.e. about 33 ms per frame)? Was some sort of regularization used? If so, which? It is not described in the methods.

Response: We apologize for not having explained the smoothing process. In calculating the BTA, the average of the preceding sequences was smoothed using temporal averaging with a 16 ms window. This smoothing removed high-frequency noise introduced by the discrete changes in the light intensity of the stimulus. Because this window was much shorter than the temporal resolution of the stimulus, the smoothing did not have a discernable effect on the overall shape of the BTAs. We now detail the smoothing process in the revised Materials and Methods ("Burst-triggered average (BTA)," "Deviation from 2-BTA," "Deviation from 3-BTA," and

"Approximately independent components").

o 2A, 3E, 5F, If you really want to know whether "the number of spikes within a burst encodes the amplitude of ON-to-OFF light intensity modulation", you need to normalize the BTAs and calculate the projection values of burst-triggered versus random-triggered events (see for instance figure 1C of Slee, S. J., Higgs, M. H., Fairhall, A. L., & Spain, W. J. (2005). Two-Dimensional Time Coding in the Auditory Brainstem. The Journal of Neuroscience€i0, 25€i0(43), 9978–9988.)

Response: We appreciate the reviewer' suggestion to analyze the projection values. The revised manuscript calculates the stimulus values projected onto the BTAs. In accordance with Slee *et al*., 2005, we normalized the prior distribution of the projection values (please see below). This normalization effectively generates the same results as the BTA normalization.

<u>Spike number (Revised Fig 2C, 2D and "Projection onto 3-BTA－1-BTA" in the revised Materials and Methods).</u>

To analyze information coding by the spike number, we projected the stimulus onto 3-BTA−1-BTA of each cell. Revised Fig 2C shows the probability density distribution of the projection values for all stimuli (dashed line) and those triggered by isolated spikes, 2-spike bursts, 3-spike bursts, and 4-spike bursts (solid lines). The projection values triggered by bursts with a different number of spikes had different probability distributions. Moreover, this shift was larger for bursts with more spikes. This result indicates that the spike number conveys information about the stimulus feature represented by 3-BTA−1-BTA.

The above analysis compares projection values at randomly sampled time points and those sampled at bursts with *k*-spikes. Thus, the difference in the probability distribution represents two pieces of information: the information conveyed by a burst occurrence (including isolated spikes) and the information conveyed by the spike number. To visually conceptualize the latter, we collected the projection values at the time point of all bursts and isolated spikes and normalized them so that the the distribution had a mean = 0 and a SD = 1. (Fig 2D, dashed line). The values for bursts with a specific number of spikes show different distributions than the values combined for all bursts and isolated spikes (Fig 2D). This result also demonstrates that the probability distribution of the projection values changes according to the spike number (Fig 2D), confirming that the spike number conveys information about the stimulus feature represented by the 3-BTA－1-BTA.

Two-spike burst ISI (Revised Fig 3I, 3J and "Deviation from 2-BTA" in the revised Materials and Methods)

We also conducted projection analysis for 2-spike burst ISIs. The stimulus feature was the difference between the oscillatory features shown by the yellow and blue lines in revised Fig 3G calculated for each cell. We collected the projection values at the time points of all 2-spike bursts and normalized the values. We then identified bursts with the shortest (0%–10%), intermediate (45%–55%), and longest (90%–100%) ISIs and collected the projection values at the time points of those bursts. The probability density distribution was determined for these values and the mean ± SEM calculated for the 41 cells is shown in Fig 3I. The probability distributions for the shortest and longest ISIs were shifted from the distribution for all 2-spike bursts combined and the shifts were in the opposite directions. This indicates that the ISIs convey information about the stimulus feature represented by the BTA.

We next conducted the same analysis but this time projecting the stimulus onto the 3-BTA − 1-BTA, which is the stimulus feature encoded by the spike number. Interestingly, 2-spike bursts with the shortest, intermediate, and longest ISIs all had projection value distributions that were almost indistinguishable from the distribution for all 2-spike bursts combined (revised Fig 3J). This result suggests that 2-spike burst ISIs convey little to no information about the stimulus feature encoded by the spike number. This result further suggests that 2-spike burst ISIs and the spike number encode different feature about the stimulus. Therefore, the result is consistent with the data showing that 2-spike burst ISIs are not correlated with the average spike number in each event.

Three-spike burst patterns (Revised Fig 7H–7K and "Approximately independent components" in the revised Materials and Methods)

To analyze the projection values for the three-spike bursts including those used in Fig 5F, we have performed the following analysis and presented the results in Fig 7H–7K. The oscillatory stimulus features $f_1(t)$ and $f_2(t)$ corresponding to the two approximately independent components w_1 and w_2 were determined as explained in the Materials and Methods.

In Fig 7H, $f_1(t)$ was used. The probability density distribution was calculated for all 3-spike bursts and for 3-spike bursts with small ($w_1 < -2$), intermediate ($-0.75 < w_1 < 0.75$), and large ($2 < w_1$) w_1 values (note that w_1 has a mean ~ 0 and SD ~ 1). The projection values collected for 3-spike bursts with small and large w_1 had probability distributions that were shifted to the left and right of that calculated for all 3-spike bursts, respectively (Fig 7H). Thus, the coordinate w_1

conveys information regarding the stimulus features represented by $f_1(t)$. We obtained similar results from the analyses for w_2 and $f_2(t)$ (Fig 7K), indicating that the coordinate w_2 conveys information regarding the stimulus features represented by $f_2(t)$. The shifts in probability density distributions were larger for w_1 (Fig 7H and 7K) which is consistent with the analysis showing that w_1 is more informative than w_2.

To analyze the specificity of information coding, we projected the stimulus onto $f_2(t)$ and collected the value at 3-spike bursts with small, intermediate, and large w_1. Interestingly, the projection values triggered by different w_1 values had probability density distributions that were indistinguishable from all 3-spike bursts combined (Fig 7I). This result suggests that the w_1 value conveys little to no information about the stimulus feature represented by $f_2(t)$. Similar results were obtained for the analyses using w_2 and $f_1(t)$ (Fig 7J). Thus, w_1 and w_2 specifically convey information about the stimulus features represented by $f_1(t)$ and $f_2(t)$, respectively. This is consistent with the idea that w_1 and w_2 encode independent information.

In sum, the BTA projection analyses indicate that (1) the spike number within a burst encodes the stimulus feature represented by the 3-BTA − 1-BTA in Fig 2B. (2) The ISI of 2-spike bursts encodes an oscillatory stimulus feature. (3) The burst ISI and spike number convey different information about the stimulus. (4) The two independent components of the 3-spike burst patterns encode two oscillatory stimulus features in a largely independent manner.

Thus, investigating the stimulus projections strengthened our original conclusions.

o 2B, 3F&G, 5D : did you subtract the means, or on a cell-by-cell basis? How was the STD calculated? Please give a description in the methods and refer to this in the text and figure caption!

Response: We apologize for our insufficient description of these calculations. All the subtractions were done on a cell-by-cell basis. We now describe the detailed calculations in the revised "Burst-triggered average (BTA)," "Deviation from 2-BTA," and "Deviation from 3-BTA" sections of the Materials and Methods and in the figure legends. These calculations are detailed below.

<u>"Burst-triggered average (BTA)" in the revised Materials and Methods</u>

In Fig 2B, the difference between the 3-BTA and 1-BTA (3-BTA–1-BTA) was calculated on a cell-by-cell basis. The mean ± SEM of 3-BTA–1-BTA was calculated across the 41 cells at each time point from −300 ms to 0 ms.

“Deviation from 2-BTA” in the revised Materials and Methods

Revised Fig 3F and 3G (Fig 3E and 3F in the original manuscript) show data from a single cell. The 2-BTA was first calculated for the cell (black in revised Fig 3F and original Fig 3E). Next, the 2-spike bursts of the cell with the longest ISIs (top 50%) were collected and the stimulus sequences preceding these bursts were determined. The 2-BTA of the cell was subtracted from these sequences, and the mean ± SEM were calculated across the sequences and are shown as thick and thin yellow lines in revised Fig 3G (original Fig 3F). The stimulus sequences preceding the 2-spike bursts with the shortest ISIs (bottom 50%) were also processed and shown as the thick and thin blue lines in revised Fig 3G (original Fig 3F). These sequences shown as thick yellow and blue lines in revised Fig 3G (original Fig 3F; termed the deviations from 2-BTA) were similarly calculated for all 41 cells. The mean ± SEM of these 41 sequences were calculated and shown in the thick and thin lines in the revised Fig 3H (original Fig 3G).

Original Figure 5

Original Fig 5 was generated using the following steps. First, for each cell, the burst phase was defined for each 3-spike burst (Fig 4E). For each phase $\theta = 0°, 1°, 2°, \cdots, 359°$, bursts with a phase larger than $\theta - 30°$ and smaller that $\theta + 30°$ were identified, and the stimulus sequences preceding these bursts were averaged. Original Fig 5A shows these 360 sequences calculated for the cell used in Fig 1 (“0” on the horizontal axis in original Fig 5A indicates the time of the first spike in the burst). The 3-BTA was also calculated for each cell (original Fig 5B). The 3-BTA was subtracted from the 360 sequences in original Fig 5A, and the resultant 360 sequences were shown in original Fig 5C. The 360 sequences similar to Fig 5C were obtained for all cells and averaged across cells to generate original Fig 5D.

Revised Fig 5 and “Deviation from 3-BTA” in the revised Materials and Methods

Because the 60° window width was a bit too large and simple averaging is easier to interpret, we changed the reporting method to simple binning and averaging, as reported in “Deviation from 3-BTA” in the revised Materials and Methods section. For each cell, three-spike bursts are divided into 12 groups according to the burst phase. The lower and upper phase limits for the bins are $0° \pm 15°, 30° \pm 15°, \cdots, 330° \pm 15°$. The preceding stimulus sequences are collected and averaged for each of the 12 burst groups. The 12 average sequences are now shown in the revised Fig 5A. The 3-BTA is also calculated for each cell (revised Fig 5B). The 3-BTA is subtracted from the 12 sequences in revised Fig 5A, and the resultant 12 sequences are shown in revised Fig 5C. The 12 sequences corresponding to Fig 5C are obtained for all cells and averaged

across cells to generate revised Fig 5D. Fig 5H is similarly revised, with 5E and 5I revised accordingly. The results in the revised figures are very similar to what we originally reported, indicating that the revision had little to no effect on the results.

• Figure 5

o This is one of the most important claims of the paper: the fast that the BTA is phase-shifted depending on the ISIs. However, this claim depends critically on how bursts of different duration are aligned (first spike, last spike, middle spike, middle of the total duration). So could the authors explain how these bursts are aligned (i.e. how the coordinate transformation affect alignment), and argue why the results in fig. 5 E&G is not simply an effect of burst alignment?

[So could the authors explain how these bursts are aligned *(i.e. how the coordinate transformation affect alignment)*]

Response: We apologize for the insufficient description. As we now describe in the revised Material and Methods section titled "Deviation from 3-BTA," bursts were aligned to the first spikes. Thus, in Fig 5 (except panel 5F), the "0" on the horizontal axis indicates the time of the first spike in each burst. Therefore, the times on the horizontal axes are the times relative to the first spike (i.e., the burst onset). The coordinate transformation defines the coordinate variables for each burst but does not affect the alignment. For example, in revised Fig 5A, 5C, 5D, and 5H, each row corresponds to bursts with phases within the same 30° bin. Thus, the coordinate transformation affects how the bursts are grouped into the rows but does not affect the alignment in the horizontal axes. We have changed the labels of the horizontal axes from "Time relative to burst onset" to "Time relative to first spike in burst" to clarify how the bursts are aligned.

[argue why the results in fig. 5 E&G is not simply an effect of burst alignment?]

Response: To evaluate the potential effect of burst alignment, we conducted the same analysis that was used for Fig 5, except that the bursts are aligned to the second spike, third spike, and middle of the total duration (S3A–S3I Fig). The results (S3A–S3I Fig) are similar to those found when aligning to the first spike (Fig 5A, 5D, and 5E). The stimulus sequences preceding the bursts (S3A, S3D, and S3G Fig) were dependent on the burst phase in a manner similar to Fig 5A. The deviation from 3-BTA also showed a similar burst phase dependence (comparingS3B, S3E, and S3H Fig to Fig. 5D). Although the deviation sequences were slightly distorted (comparingS3C, S3F, and S3I Fig to Fig. 5E), they were all oscillatory sequences. Finally, their peaks (allows inS3C, S3F, and S3I Fig) had temporal shifts like those shown in Fig 5E. These results indicate that aligning the burst to the first, second, and third spikes and the middle of the

total duration produced similar results. In sum, these results suggest that the data shown in Fig 5E are not simply an effect of burst alignment. We discuss this issue in the last paragraph of "The two ISIs of three-spike bursts encode the phase and amplitude of oscillatory components" in Results.

We apologize for the insufficient description for Fig 5G. This figure shows a reconstruction of stimulus sequences preceding a simulated burst, which is represented by the vertical lines as described in the revised Material and Methods section titled “Linear reconstruction.” Because this figure only characterizes one burst, no burst alignment was performed. Therefore, burst alignment is irrelevant to Fig 5G.

Fig 5H and 5I show an analysis of the same bursts in Fig 5A and 5E, respectively. The same alignment to the first spike was used for Fig 5A, 5E, 5H, and 5I. For Fig 5H and 5I, the preceding stimulus sequences were replaced with the reconstructed sequences. Thus, burst alignment cannot be a factor for the difference between Fig 5A and 5H or the difference between 5E and 5I.

o G-I: the explanation in the caption is not enough to follow what is going on here. Please explain what is done and why. Also, these are not referred to in the main text, please add it to the general story.

Response: We apologize for the unclear caption in the original manuscript. We have revised the caption for Fig 5G–5I, provided the Materials and Methods section “Linear reconstruction,” and devoted a result section titled “Linear reconstruction fails to decode the burst pattern” to discuss the results in the context of decoding.

• Figure 6

o See figure 5: explain burst alignment.

Response: We apologize for the insufficient description of the burst alignment. Similar to Fig 5, the bursts in Fig 6B–6D were aligned to their first spike, indicated as “0” on the horizontal axes. Therefore, the time shown on the horizontal axes are relative to the first spike (i.e., the burst onset). The revised Materials and Methods section provides a mathematical expression of this alignment in the “Burst-triggered average (BTA)” and “Deviation from 3-BTA” sections. In addition, we changed the labels of the horizontal axes from “Time relative to burst onset” to “Time relative to first spike in burst.”

In Fig 6A, the bursts are aligned to the stimulus time. The “0” on the horizontal axes indicate the average time of the first burst spike in each event.

o B: the last 2 groups (6.4-14.5 ms and < 6.4 ms) have the same color in the pdf.

Response: We thank the reviewer for pointing out the ambiguous color labeling. We have changed the colors so that the lines can be clearly distinguished.

o D: it is not explained what the arrows indicate.

Response: We apologize for this mistake. The arrows indicate the first peak before the first spike in a burst. We have corrected Fig 6D legend accordingly.

Figure 7:

o C: where are the data points? Could these be added? Also, could the axis have ticks+numbers, so that B and C can be compared (I suppose the magenta line should be in the same direction)? Or alternatively, could C and D be reversed in order (then it makes a lot more sense)?

[C: where are the data points? Could these be added?]

Response: We apologize for the insufficient data in the figure. The data points have been added to revised Fig 7D. We have removed the circular histogram because the data points sufficiently demonstrate the data distribution.

o E-F: please, don't just say 'the same as some other figure', but explain, that makes it so much easier to read.

Response: We apologize for the insufficient caption. The revised caption includes the details regarding the data.

2.3 Main text

Line 134: "Two-spike burst ISIs were not correlated to the average number of spikes in events (Fig 3B…" I would expect for this claim, a plot of the ISIs on one axis, and the number of spikes in an event on the other, with a correlation measure. However, here you just see for 4 events the ISIs. So this does not show whether there is a correlation between ISIs and number of spikes or not.

Response: We apologize for our insufficient description. The original manuscript calculated the correlation between the ISIs and spike number (Lines 134–135 and 423–428 in the original manuscript). However, as indicated by the reviewer, a figure showing the relationship between the ISIs and spike number is preferable. Therefore, we added a new figure (revised Fig 3D) to the

manuscript. This figure shows the data from a single cell, with each dot representing an event. The horizontal and vertical axes are the mean intraburst ISI of 2-spike bursts and the mean spike number, respectively. The figure shows that the correlation between the intraburst ISIs and spike numbers was very weak (correlation coefficient = −0.15). For the 41 cells analyzed, the correlation coefficient was 0.0 ± 0.1 (mean ± SD). These results confirm that the burst ISIs and number of spikes have very little to no correlation.

Line 104, 147: I think for these claims, projection values need to be calculated (see for instance figure 1C of Slee, S. J., Higgs, M. H., Fairhall, A. L., & Spain, W. J. (2005). Two-Dimensional Time Coding in the Auditory Brainstem. The Journal of Neuroscience, 25(43), 9978–9988.)

Response: We appreciate the reviewer' suggestion to analyze the projection values. The revised manuscript calculates the stimulus values projected onto the BTAs. In accordance with Slee *et al.*, 2005, we normalized the prior distribution of the projection values (please see below). This normalization effectively generates the same results as the BTA normalization.

Spike number (Revised Fig 2C, 2D and "Projection onto 3-BTA－1-BTA" in the revised Materials and Methods).

To analyze information coding by the spike number, we projected the stimulus onto 3-BTA−1-BTA of each cell. Revised Fig 2C shows the probability density distribution of the projection values for all stimuli (dashed line) and those triggered by isolated spikes, 2-spike bursts, 3-spike bursts, and 4-spike bursts (solid lines). The projection values triggered by bursts with a different number of spikes had different probability distributions. Moreover, this shift was larger for bursts with more spikes. This result indicates that the spike number conveys information about the stimulus feature represented by 3-BTA−1-BTA.

The above analysis compares projection values at randomly sampled time points and those sampled at bursts with *k*-spikes. Thus, the difference in the probability distribution represents two pieces of information: the information conveyed by a burst occurrence (including isolated spikes) and the information conveyed by the spike number. To visually conceptualize the latter, we collected the projection values at the time point of all bursts and isolated spikes and normalized them so that the distribution had a mean = 0 and a SD = 1. (Fig 2D, dashed line). The values for bursts with a specific number of spikes show different distributions than the values combined for all bursts and isolated spikes (Fig 2D). This result also demonstrates that the probability distribution of the projection values changes according to the spike number (Fig 2D), confirming that the spike number conveys information about the stimulus feature represented by

the 3-BTA−1-BTA.

<u>Two-spike burst ISI (Revised Fig 3I, 3J and "Deviation from 2-BTA" in the revised Materials and Methods)</u>

We also conducted projection analysis for 2-spike burst ISIs. The stimulus feature was the difference between the oscillatory features shown by the yellow and blue lines in revised Fig 3G calculated for each cell. We collected the projection values at the time points of all 2-spike bursts and normalized the values. We then identified bursts with the shortest (0%–10%), intermediate (45%–55%), and longest (90%–100%) ISIs and collected the projection values at the time points of those bursts. The probability density distribution was determined for these values and the mean ± SEM calculated for the 41 cells is shown in Fig 3I. The probability distributions for the shortest and longest ISIs were shifted from the distribution for all 2-spike bursts combined and the shifts were in the opposite directions. This indicates that the ISIs convey information about the stimulus feature represented by the BTA.

We next conducted the same analysis but this time projecting the stimulus onto the 3-BTA−1-BTA, which is the stimulus feature encoded by the spike number. Interestingly, 2-spike bursts with the shortest, intermediate, and longest ISIs all had projection value distributions that were almost indistinguishable from the distribution for all 2-spike bursts combined (revised Fig 3J). This result suggests that 2-spike burst ISIs convey little to no information about the stimulus feature encoded by the spike number. This result further suggests that 2-spike burst ISIs and the spike number encode different feature about the stimulus. Therefore, the result is consistent with the data showing that 2-spike burst ISIs are not correlated with the average spike number in each event.

Line 180: Explain how this linear and non-linear scaling was done. There is some text in the methods, but it is not referred to, and also explain the gist of it in the main text.

Response: We apologize for the insufficient description of the coordinate transformation. We now describe the mathematical processes for the transformation in the revised method ('Coordinate transformation of 3-spike bursts') and the gist of the scaling in the main text (equations 1 and 2).

Line 183: you use u1 and u2 for both the base vectors and their components. This is unclear mathematical notation.

Response: We apologize for the ambiguous description of u_1 and u_2 as representation of two

scaler variables in the original manuscript. The revised text includes improved mathematical notations of the coordinate transformation that clearly the definition of u_1 and u_2 ("Coordinate transformation of 3-spike bursts" in Materials and Methods and equations 2 in the main text).

Line 232 and further: I get lost here: you're comparing two groups with a separation of 80 ms, but both have phase 0, so what is it that you're comparing?

Response: We apologize for the unclear explanation. The revised manuscript explains this analysis in the context of decoding, which allows us to remove these sentences. We believe that this clarifies the purpose and processes of the analyses.

After this: how was the reconstruction done exactly? I tried to infer it from the methods, but not enough detail was provided. What does "estimated stimulus sequence by adding the STA aligned according to the burst spikes" mean? Do you generate an STA for every type of burst? But what do you add then? Or do you generate an STA for a silence of length x + spike sequence?

Response: We apologize for the poor description of the reconstruction. The method was completely rewritten for the revised manuscript and includes the precise mathematical formulations ("Linear reconstruction" in Materials and Methods).

2.4 Discussion

Good that both possible encoding and decoding mechanisms are discussed! Since you mention a variation of ISI sensitivity: Did you observe neural homogeneity or heterogeneity in your recorded neurons? Did you find all neurons code for exactly the same features? In the case of 2-spike bursts, did they encode the same features at the same phase? Or was there variability between the neurons?

Response: We thank the reviewer for raising the issue of variability between the neurons. We plotted the stimulus sequences encoded by the 2-spike burst ISIs and the 2 independent components for each analyzed neuron (revised S2 Fig). The figures show that the encoded sequences were similar for most neurons (~15 of the 19 cells), indicating that most cells have similar encoding characteristics. The encoded phase is slightly varied but mostly similar for the majority of neurons. A small fraction of cells (~4 cells of the 19 cells) appeared to exhibit different patterns, suggesting some variability among neurons. We mentioned the individual cell data in the revised manuscript (the second paragraph in "Burst ISIs encode oscillatory light intensity sequences" and the third paragraph in "Two independent components of the burst patterns" in results).

2.5 Methods

Burst and event definition

• There is quite a bit difference between the threshold-ISI for bursts for the Gaussian sequence and the natural movie. Could the authors please share their thoughts on

Response: As indicated by the reviewer, the threshold-ISIs for the Gaussian sequence and the natural movie stimuli were ~40 ms and ~90 ms, respectively. Our method did not manually select these thresholds but set them at the lowest point of the trough between the two peaks observed in the ISI histogram. Therefore, the threshold-ISIs were set at ~40 ms and ~90 ms because the spike firing is correlated up to ~40 ms and ~90 ms for the Gaussian and natural movie stimuli, respectively.

On the other hand, the temporal constant $\tau_{1/2}$, which represents the half-width at half maximum of the autocorrelation, was 33 ms and 90 ms for the Gaussian and natural movie stimuli (please see the revised Materials and Methods). These values indicate that the light intensity value for each stimulus is similar for ~33 ms and ~90 ms. Therefore, spike firing elicited by the stimuli is likely correlated for ~33 ms and ~90 ms for the Gaussian and natural movie stimuli, respectively. These differences in the firing correlation would cause differences in the ISI histogram, leading to differences in the burst-threshold-ISIs. Therefore, the difference in the burst-threshold-ISIs likely reflects the difference in the temporal correlation of the stimuli. We discuss this issue in the "Generality of the combinatorial ISI coding" in revised Discussion.

We would like to note that although the threshold-ISI is ~90 ms for the natural movie, the average burst duration is much shorter (2-spike bursts: 15.4 ± 5.6 ms; 3-spike bursts: 29.0 ± 9.8 ms).

• I don't understand the event extraction, the description is not quite clear (although I do understand the gist of it). Could it please be clarified?

Response: We apologize for our insufficient description of event identification. We have used mathematical expressions to improve the description in the method ("Identification of Bursts and Events" in revised Materials and Methods) and have revised the figure (revised S1 Fig) to clarify the method.

Mutual information of spike number in a burst:

The mutual information calculation is a bit strange: the authors seem to calculate the mutual

information between the number of spikes in a burst (k) and the event number j. However, they never state this explicitly. Moreover, why is this a relevant mutual information to calculate? I would say that the receiver, i.e. the visual thalamus, cares about the stimulus, not about the event number? Maybe there is a good reason for calculating this, but the authors don't explain this.

Response: We thank the reviewer for raising the issue of information calculation. In the original manuscript, we calculated the mutual information between the number of spikes and the event identity and between the burst ISIs and the event identity. This method implicitly assumed that information about the event identity was similar to information about the stimulus. However, this assumption is not theoretically guaranteed. Indeed, information values calculated in this manner could have a complicated relationship with the stimulus information. First, repeating the stimulus causes bursts in the same event to occur at a slightly different time relative to the stimulus. This timing jitter affects the amount of information that can be obtained regarding the stimulus by knowing the event identity. Moreover, different events can have different jitters that have varied effects on the information about the stimulus. Second, if the spike number and/or ISIs of consecutive events are not independent, the event identity information can have a complicated relationship with information about the stimulus.

Therefore, the revised manuscript utilizes a method that does not rely on hypotheses about encoding and provides a better estimation of information about the stimulus (Brenner et al., Synergy in a Neural Code, Neural Computation 12, 1531, 2000; "Mutual information" in revised Materials and Methods). The revised information values tended to be larger than those calculated in the original analysis and supported our previous conclusions. We apologize for our original inaccurate calculation.

A single spike is counted as a burst??

Response: We apologize for the unclear description. Ganglion cells often generate single spikes that are preceded and followed by intervals longer than T_{thresh}. We designate these spikes "isolated spikes" (revised S1C Fig legend). Isolated spikes can occur in events that have bursts with more than one spikes (e.g., revised Fig 1, purple). Therefore, isolated spikes are essentially similar to bursts with more than one spikes, and thus we treated them as "1-spike" bursts. This definition is a natural generalization of that of bursts and enables us to use the same method for isolated spikes and bursts with ≥ 2 spikes (e.g., Fig 2).

Mutual information of ISIs:

The fact that you divide the ISIs in four groups of equal size, means that you exclude the option

of 'rare but very informative events' occurring. Could the authors explain why they made this decision?

Response: We thank the reviewer for pointing out the issue of information calculation. We implemented a multi-step process that contributed to dividing the ISIs into four groups. We first performed a simulation that assumed that the ISIs occur according to a distribution that the mutual information can theoretically be derive from. We then generated ISIs according to this distribution and divided them into 3-6 groups before calculating mutual information. The result showed that dividing into four groups gave the most accurate estimation. Therefore, we decided to divide the ISIs into four groups.

However, the above method relies on an artificial distribution and thus it is not guaranteed to be suitable for the real data. Thus, we changed the information calculation method in the revised manuscript. In the revised method, we tested multiple ISI bin sizes (Δt) and estimated the value at the limit $\Delta t \rightarrow 0$. We describe this improvement in the revised Materials and Methods ("Burst-triggered average (BTA)").

Coordinate transformations

I understand the gist of what you do, but again, some information is lacking.

Response: We apologize for the insufficient description of the coordinate transformation. Revised Materials and Methods now describes the mathematical processes for the transformation ("Coordinate transformation of 3-spike bursts") and the method is briefly explained in the main text (equations 1 and 2).

Why were events with small ISI excluded? Doesn't this bias the results?

[Why were events with small ISI excluded?]

Response: For a 3-spike burst with interspike intervals ISI_1 and ISI_2, the variables v_1 and v_2 were determined as

$$v_1 = \log_{10}(\mathrm{ISI}_1[\mathrm{ms}] - m_1[\mathrm{ms}])$$

$$\text{and } v_2 = \log_{10}(\mathrm{ISI}_2[\mathrm{ms}] - m_2[\mathrm{ms}]),$$

where m_1 and m_2 are the intersects determined in Fig 4C. Because $\log_{10}(x)$ cannot be defined for $x \leq 0$, v_1 cannot be defined for bursts with $\mathrm{ISI}_1 \leq m_1$ and v_2 cannot be defined for bursts with $\mathrm{ISI}_2 \leq m_2$. We have thus excluded bursts with $\mathrm{ISI}_1 \leq m_1$ or $\mathrm{ISI}_2 \leq m_2$. We

explained the exclusion in the revised method ("Coordinate transformation of 3-spike bursts") and text (the last paragraph in "The two ISIs of three-spike bursts encode the phase and amplitude of oscillatory components" in the main text).

[Doesn't this bias the results?]

Response: Our analyses show that bursts with $\mathrm{ISI}_1 > m_1$ and $\mathrm{ISI}_2 > m_2$ contain information as it is described in the manuscript. Therefore, if the brain behaves as an ideal decoder, it can select those bursts and extract the information without a bias. To further evaluate the stimulus coding with bursts with small ISIs we tested a new decoding scheme that includes bursts with small ISIs. For each cell we collected all bursts that were not excluded. Among these bursts, we determined the smallest $\mathrm{ISI}_1(> m_1)$ and smallest $\mathrm{ISI}_2(> m_2)$, which were termed ISI_{10} and ISI_{20}, respectively. We then exchanged the ISIs of all the excluded bursts with ISI_{10} and ISI_{20} and incorporated them into the analysis. The results were almost indistinguishable from the original results (comparing S3M–S3O Fig with Fig 5A, 5D, and 5E), indicating that excluding bursts with small ISIs had a minimal effect on the analysis. This result suggests that encoding by bursts with very short ISIs is similar to that for other bursts. We have described the method in the "Test of the effect of removing 3-spike bursts with short ISIs" section in revised Materials and Methods and discussed it in the last paragraph of "The two ISIs of three-spike bursts encode the phase and amplitude of oscillatory components" in the main text.

In addition to the requested revision, we have made several changes in the revision. In the coordinate transformation of 3-spike burst ISIs, variables are normalized before the principle component analysis, but we failed to explain this normalization in the original manuscript. In addition, we found that the normalization used in original Figs 6D and 7 was different from that for Fig 5. In the revised manuscript we have described the normalization, removed the principle axes in Fig 5D, and performed the analysis in Figs 6D and 7 using the same normalization that is used in Fig 5. Revised Fig 6 presenting the natural scene experiment shows that the burst phase encodes the temporal phase of the deviation from 3-BTA like the original manuscript. However, the correlation appears to be weaker than that in the original analysis (revised Fig 6D). We consider this issue in the discussion section ("Generality of the combinatorial ISI coding"). Revised Fig 7 is very similar to original Fig 7. The orientation of the steepest gradient in Fig 7B (original: 33.1° ± 15.6°, revised: 32.3° ± 15.6°; Fig 7D, magenta) and the orientation of the principle axes in Fig 7C (original: 29.4° ± 7.3° and 119.4° ± 7.3°, revised: 28.6° ± 7.7° and 118.6° ± 7.7°, respectively; Fig 7D, yellow and green, respectively) have been revised accordingly in the main text. We apologize for the initial issue with the coordinate transformation.

In addition, in Figs 4B, 4D, 4E, 7C, 7E, and 7G, ~1.5% of data points are out of the range of the plot. We now describe this issue in the figure captions.

We have answered the reviewer's comments made in the PDF file of the original manuscript.

Finally, we would like to thank the reviewer for the numerous thoughtful suggestions, which greatly improved the scientific quality of the manuscript.

PLOS Computational Biology

Interspike intervals within retinal spike bursts combinatorially encode multiple stimulus features

--Manuscript Draft--

**Manuscript Number:**	PCOMPBIOL-D-20-00205
**Full Title:**	Interspike intervals within retinal spike bursts combinatorially encode multiple stimulus features
**Short Title:**	Burst interspike interval coding in the retina
**Article Type:**	Research Article
**Keywords:**	retina; retinal ganglion cells; coding; spike bursts; combinatorial coding
**Abstract:**	Neurons in various regions of the brain generate spike bursts. While the number of spikes within a burst has been shown to carry information, information coding by interspike intervals (ISIs) is less well understood. In particular, a burst with k spikes has $k-1$ intrabust ISIs, and these $k-1$ ISIs could theoretically encode $k-1$ independent values. In this study, we demonstrate that such combinatorial coding occurs for retinal bursts. By recording ganglion cell spikes from isolated salamander retinae, we found that intraburst ISIs encode oscillatory light sequences that are much faster than the light intensity modulation encoded by the number of spikes. When a burst has three spikes, the two intraburst ISIs combinatorially encode the amplitude and phase of the oscillatory sequence. Analysis of trial-to-trial variability suggested that intraburst ISIs are regulated by two independent mechanisms responding to orthogonal oscillatory components, one of which is common to bursts with different number of spikes. Therefore, the retina encodes multiple stimulus features by exploiting all degrees of freedom of burst spike patterns, i.e., the spike number and multiple intraburst ISIs.
**Additional Information:**	[illegible]
**Question**	[illegible]
**Financial Disclosure**<br><br>Enter a financial disclosure statem[illegible] describes the sources of funding fo[illegible] work included in this submission. R[illegible] the submission guidelines for detailed requirements. View published research articles from *PLOS Computational Biology* for specific examples.<br><br>This statement is required for submission and **will appear in the published article** if the submission is accepted. Please make sure it is accurate.	[illegible] to T.H. and Grant-in-Aid for [illegible]re, Sports, Science and [illegible]unders had no role in study [illegible] or preparation of the

These pages are NOT the revised manuscript, but the original manuscript. They are provided to answer the reviewer's comments written in the PDF file of the original manuscript.

Unfunded studies
Enter: *The author(s) received no specific funding for this work.*

Funded studies
Enter a statement with the following details:
- Initials of the authors who received each award
- Grant numbers awarded to each author
- The full name of each funder
- URL of each funder website
- Did the sponsors or funders play any role in the study design, data collection and analysis, decision to publish, or preparation of the manuscript?
- **NO** - Include this sentence at the end of your statement: *The funders had no role in study design, data collection and analysis, decision to publish, or preparation of the manuscript.*
- **YES** - Specify the role(s) played.

* typeset

These pages are NOT the revised manuscript, but the original manuscript. They are provided to answer the reviewer's comments written in the PDF file of the original manuscript.

Competing Interests | [illegible]exist.

Use the instructions below to ente[illegible] competing interest statement for t[illegible] submission. On behalf of all autho[illegible] disclose any competing interests that could be perceived to bias this work—acknowledging all financial support and any other relevant financial or non-financial competing interests.

This statement **will appear in the published article** if the submission is accepted. Please make sure it is accurate. View published research articles from *PLOS Computational Biology* for specific examples.

Powered by Editorial Manager® and ProduXion Manager® from Aries Systems Corporation

NO authors have competing interests

Enter: *The authors have declared that no competing interests exist.*

Authors with competing interests

Enter competing interest details beginning with this statement:

I have read the journal's policy and the authors of this manuscript have the following competing interests: [insert competing interests here]

* typeset

Data Availability

These pages are NOT the revised manuscript, but the original manuscript. They are provided to answer the reviewer's comments written in the PDF file of the original manuscript.

Authors are required to make all data [illegible] underlying the findings described full[illegible] available, without restriction, and fro[illegible] time of publication. PLOS allows rare [illegible] exceptions to address legal and ethi[illegible] concerns. See the PLOS Data Policy [illegible] FAQ for detailed information.

A Data Availability Statement describing where the data can be found is required at submission. Your answers to this question constitute the Data Availability Statement and **will be published in the article**, if accepted.

Important: Stating 'data available on request from the author' is not sufficient. If your data are only available upon request, select 'No' for the first question and explain your exceptional situation in the text box.

Do the authors confirm that all data underlying the findings described in their manuscript are fully available without restriction?

**Describe where the data may be found in full sentences. If you are copying our sample text, replace any instances of XXX with the appropriate details.**	All data files are available at: https://osf.io/29ect/.

Powered by Editorial Manager® and ProduXion Manager® from Aries Systems Corporation

• If the data are **held or will be held in a public repository**, include URLs, accession numbers or DOIs. If this information will only be available after acceptance, indicate this by ticking the box below. For example: *All XXX files are available from the XXX database (accession number(s) XXX, XXX.)*.<br>• If the data are all contained **within the manuscript and/or Supporting Information files**, enter the following: *All relevant data are within the manuscript and its Supporting Information files.*<br>• If neither of these applies but you are able to provide **details of access elsewhere**, with or without limitations, please do so. For example:<br><br>*Data cannot be shared publicly because of [XXX]. Data are available fro[illegible] XXX Institutional Data Access / [illegible] Committee (contact via XXX) fo[illegible] researchers who meet the crite[illegible] access to confidential data.*<br><br>*The data underlying the results [illegible] presented in the study are avail[illegible] from (include the name of the th[illegible] and contact information or URL).*<br>• This text is appropriate if the data are owned by a third party and authors do not have permission to share the data.<br><br>* typeset	
Additional data availability information:	

These pages are NOT the revised manuscript, but the original manuscript. They are provided to answer the reviewer's comments written in the PDF file of the original manuscript.

Manuscript
Click here to download Manuscript Ishii_200209_2100.pdf

Interspike intervals within retinal spike bursts combinatorially encode multiple stimulus features

Toshiyuki Ishii[1,2,3] and Toshihiko Hosoya[1#*]

These pages are NOT the revised manuscript, but the original manuscript. They are provided to answer the reviewer's comments written in the PDF file of the original manuscript.

[1] RIKEN Center for Br[illegible]o-shi, Saitama 351-0198, Japan.

[2] Toho University, Fun[illegible], Japan.

[3] Department of Physiology, Nippon Medical School, Bunkyo-ku, Tokyo 113-8602, Japan.

[#] Current address: Biomedical Research Department, Ricoh Company Ltd, Kawasaki-shi, Kanagawa 210-0821, Japan.

[*] **Corresponding author:**

E-mail: toshihiko.hosoya@jp.ricoh.com.

These pages are NOT the revised manuscript, but the original manuscript. They are provided to answer the reviewer's comments written in the PDF file of the original manuscript.

Abstract

Neurons in various regions of the brain generate spike bursts. While the number of spikes within a burst has been shown to carry information, information coding by interspike intervals (ISIs) is less well understood. In particular, a burst with k spikes has $k-1$ intraburst ISIs, and these $k-1$ ISIs could theoreticall[illegible]monstrate that such combinatorial coding [illegible]pikes from isolated salamander retinae, w[illegible]quences that are much faster than the [illegible]pikes. When a burst has three spikes, the t[illegible]de and phase of the oscillatory sequence. Analysis of trial-to-trial variability suggested that intraburst ISIs are regulated by two independent mechanisms responding to orthogonal oscillatory components, one of which is common to bursts with different number of spikes. Therefore, the retina encodes multiple stimulus features by exploiting all degrees of freedom of burst spike patterns, i.e., the spike number and multiple intraburst ISIs.

Author Summary

Neurons in various regions of the brain generate spike bursts. Bursts are typically composed of a few spikes generated within dozens of milliseconds, and individual bursts are separated by much longer periods of silence (~hundreds of milliseconds). Recent evidence indicates that the number of spikes in a burst, the interspike intervals (ISIs), and the overall duration of a burst, as well as the timing of burst onset, encode information. However, it remains unknown whether multiple ISIs within a single burst encode multiple independent information contents. Here we demonstrate that such combinatorial ISI coding occurs for spike bursts in the retina. We recorded

ganglion cell spikes from isolated salamander retinae stimulated with computer-generated movies. Visual response analyses indicated that multiple ISIs within a single burst combinatorially encode the phase and amplitude of oscillatory light sequences, which are different from the stimulus feature encoded by the spike number. The result demonstrates that the retina encodes mul[illegible]edom of burst spike patterns, i.e., the s[illegible]aptic transmission in the visual system is hi[illegible]ust have a major impact on visual informat[illegible]

These pages are NOT the revised manuscript, but the original manuscript. They are provided to answer the reviewer's comments written in the PDF file of the original manuscript.

Introduction

Understanding the rules by which neuronal spike patterns encode information is essential for investigating the complex functioning of the nervous system [1, 2]. Neurons in various brain areas generate spike bursts, which are characterized by clusters of high-frequency spikes separated by longer periods of silence [3-5]. Burst spikes typically occur within the temporal window of postsynaptic integration (dozens of milliseconds), and thereby i[illegible]lucing synaptic response wit[illegible]gher probability than isolated single spikes [6-8]. In this regard, bursts are believed to represent an important neuronal code [7, 9, 10]. Previous analyses of burst information coding have suggested that the number of spikes within a burst [4, 11-19], the onset timing of a burst [5, 6, 20-22], and the duration of a burst [15, 23] all carry information.

Because a burst has multiple spikes, it has one or more intraburst interspike intervals (ISIs). In theory, these intraburst ISIs can carry information if, for example, they are modulated by sensory inputs. Such burst ISI coding should have significant effects on information transfer, because the efficiency of synaptic transmission is sensitive to ISIs [24]. Consistent with this idea,

recent studies suggest that ISIs within bursts carry information [15, 19, 23, 25]. Although these studies have shown that the first ISI and average ISI within a burst carry information, interaction among multiple ISIs has been unclear. Theoretically, bursts with k spikes have k−1 intraburst ISIs, and these k−1 ISIs could encode k−1 independent values that represent information. Whether burst ISIs encode information in such a combinatorial manner is unknown.

In the vertebrate retina, retinal ganglion cells (i.e., the output neurons) generate spike bursts [3, 4, 26]. While the number of spikes within bursts encodes the amplitude of light intensity modulation [4], it is unknown whether intraburst ISIs encode information. In this study, using isolated salamander retinae, we investigated whether intraburst ISIs encode information regarding visual input. Our results indicated that intraburst ISIs encode oscillatory light intensity sequences different from the stimulus feature encoded by the spike number. When bursts contained three spikes, the two ISIs combinatorially encoded the amplitude and phase of the oscillatory componen[illegible]ested that intraburst ISIs are determined by [illegible] to two orthogonal oscillatory componen[illegible]e ISIs within a retinal burst combinatorially [illegible]e different from that encoded by the spike number.

These pages are NOT the revised manuscript, but the original manuscript. They are provided to answer the reviewer's comments written in the PDF file of the original manuscript.

Results

Burst spike numbers encode the amplitude of light intensity modulation

We stimulated isolated larval salamander retinae using a spatially uniform visual stimulus with intensity modulation set at 30 Hz. Ganglion cell action potentials were recorded using a multi-electrode array. OFF ganglion cells, constituting the majority of the larval salamander retinal

ganglion cells, generated spike bursts (Fig 1A). The majority of the spikes [82.0% ± 8.7%, mean ± SD (standard deviation), n = 41 cells] were observed in bursts comprised of two or more spikes, indicating that multi-spike bursts represent the major retinal code.

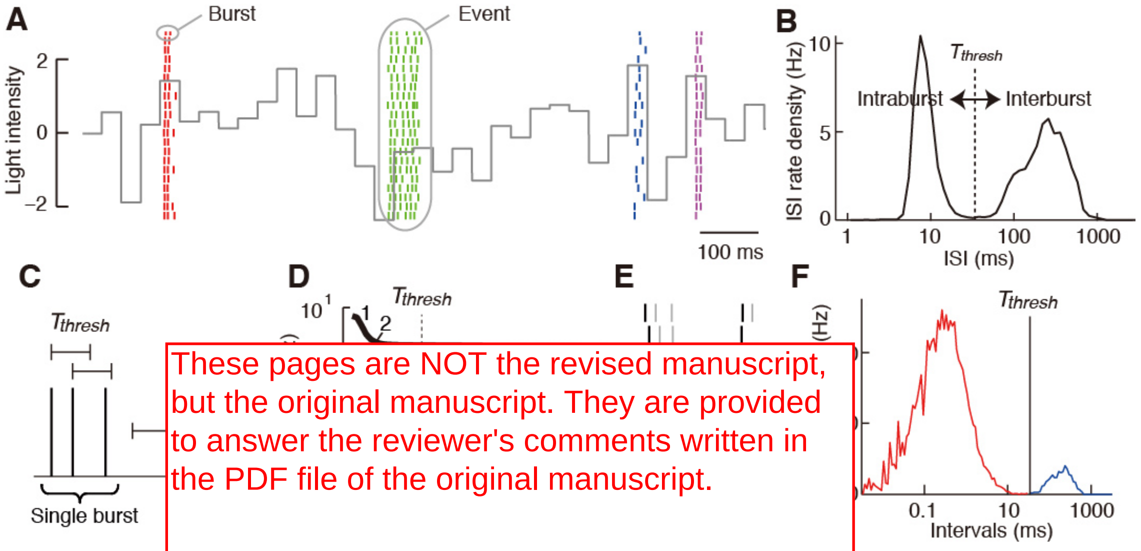

These pages are NOT the revised manuscript, but the original manuscript. They are provided to answer the reviewer's comments written in the PDF file of the original manuscript.

Fig 1. Retinal ga[illegible]n a single salamander OFF ganglion cell. **(A)** Raster plot. Short vertical lines represent single spikes and each row shows spikes that occurred during a single repeat of the stimulation. Spikes of different events are shown in different colors. The gray continuous line shows the normalized light intensity of the stimulus (the mean and SD are 0 and 1, respectively). **(B)** ISI histogram. **(C)** Schematic illustration of the algorithm to defin[illegible]ursts. **(D)** Rates of isolated spikes (1) and bursts with 2–7 spikes (2–7) plotted against the threshold interval. **(E)** Schematic illustrati[illegible]f the algorithm to define events. **(F)** Histogram of intervals between merged onsets. The red and blue portions indicate intervals shorter and longer than T_{thresh}, respectively.

During repeated presentation of the same stimulus, individual ganglion cells generated spike bursts at similar time-points across repeats (Fig 1A) [3, 4]. This reproducibility enabled the identification of corresponding bursts across repeats, which we termed “events” (Fig 1 and Materials and Methods) [3, 4]. Bursts generated in the same event had similar numbers of spikes in different repeats of [illegible] d different numbers of spikes (Fig 1A). Acc[illegible] formation about the stimulus ($p < 0.01$ fo[illegible] was 0.80 ± 0.31 bits per burst, mean ± SD [illegible] ges (BTAs), which represent the average [illegible] ts with two, three, and four spikes (1-, 2-, 3-, and 4-BTA, respectively). The BTAs were sequences of different amplitudes (Fig 2A), and the difference between 1-BTA and 3-BTA had ON and OFF peaks around −170 and −40 ms relative to the burst onset (Fig 2B). This result indicates that the number of spikes within a burst encodes the amplitude of ON-to-OFF light intensity modulation within an interval of ~130 ms.

These pages are NOT the revised manuscript, but the original manuscript. They are provided to answer the reviewer's comments written in the PDF file of the original manuscript.

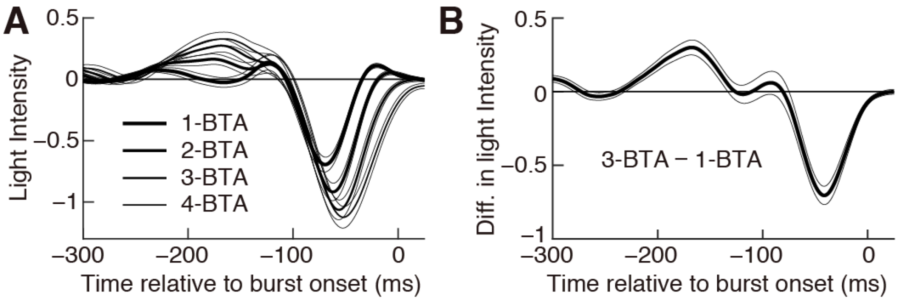

Fig 2. Spike number within a burst encodes the amplitude of light intensity modulation. (**A**) 1-, 2-, 3-, and 4-BTA indicate the averages of all stimulus sequences preceding isolated spikes, 2-, 3-, and 4-spike bursts, respectively. (**B**) Difference between 3-BTA and 1-BTA. Thick and thin lines indicate the average and standard error of mean (SEM) values, respectively. $n = 41$ cells.

Burst ISIs encode oscillatory light intensity sequences

To investigate whether intraburst ISIs carry information, we first analyzed bursts composed of two spikes (2-spike bursts). For each ganglion cell, 2-spike bursts in the same event tended to have similar ISIs, whereas those in different events typically had different ISIs (Fig 3A). This suggested that intraburst ISIs convey information about the stimulus. Calculation of the mutual information confirmed this notion ($p < 0.01$ for 41 of the 41 cells; 0.33 ± 0.10 bits per burst, n = 41).

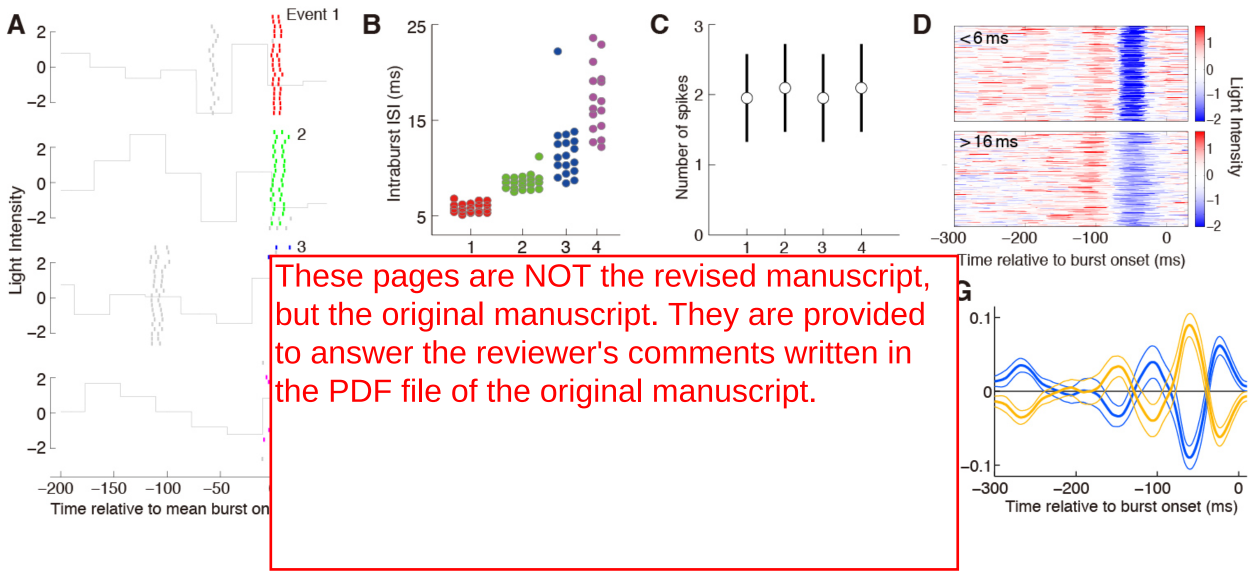

These pages are NOT the revised manuscript, but the original manuscript. They are provided to answer the reviewer's comments written in the PDF file of the original manuscript.

Fig 3. Intraburst ISIs of 2-spike bursts encode an oscillatory component of the visual input. (A-F) Data from the cell shown in Fig 1. **(A)** Raster plot. Colored lines represent 2-spike bursts. Short gray lines are the other spikes. The gray continuous line indicates light intensity, as shown in Fig 1A. Event IDs are shown. **(B and C)** Intraburst ISIs of 2-spike bursts (B) and the average and SD of the spike number (C) of the four events shown in (A). **(D)** Stimulus sequences preceding 2-spike bursts with an intraburst ISI of <6.0 (top) and >16.0 ms (bottom) are aligned with respect to the burst onset. **(E)** The thick black line indicates the average of all stimulus sequences preceding 2-spike bursts (2-BTA). Thick colored lines indicate the average of stimulus

sequences preceding 2-spike bursts with different intraburst ISIs. Thin lines indicate SEM values. **(F)** Thick yellow and blue lines indicate the average of the stimulus sequence preceding 2-spike bursts with the longest and shortest 50% of intraburst ISIs, respectively, from which the 2-BTA is subtracted. The thin lines indicate SEM values. **(G)** Population analysis. Thick lines indicate the data shown by the thick lines in (F) averaged among 19 cells that generated at least 1500 2-spike bursts. Thin lines represent SEM values.

Two-spike burst ISIs were not correlated to the average number of spikes in events (Fig 2B and C; correlation = 0.0 ± 0.1, mean ± SD, n = 41), suggesting that these ISIs were modulated according to stimulus features different from those modulating the spike number. To characterize the stimulus featur[illegible]ulus sequences preceding 2-spike b[illegible]how that the stimulus sequences had syst[illegible]btracted the 2-BTA (black in Fig 3E) fr[illegible] with long ISIs. The result was an oscillating sequence with two ON and two OFF peaks (yellow in Fig 3F and G). The subtraction from the 2-spike bursts with short ISIs gave the same sequence, but with the opposite sign (blue in Fig 3F and G). These results indicate that 2-spike burst ISIs encode an oscillatory deviation from the 2-BTA. Intervals between the ON and OFF peaks were ~40 ms (Fig 3F and G) and, therefore, were much shorter than those of the stimulus feature encoded by the spike number (~130 ms; Fig 2B). Thus, two-spike bu[illegible]SIs encode oscillatory sequences much faster than the intensity modulation encoded by the spike number.

These pages are NOT the revised manuscript, but the original manuscript. They are provided to answer the reviewer's comments written in the PDF file of the original manuscript.

The two ISIs of three-spike bursts encode the phase and amplitude of oscillatory components

We next investigated the characteristics of 3-spike bursts. The first and second intraburst ISIs (ISI_1 and ISI_2) tended to be different for different events (Fig 4A and B) and carried information about the stimulus ($p < 0.01$ for 40 of the 41 cells; 0.46 ± 0.21 bits per burst, $n = 41$). In addition, the data suggest that ISI_1 and ISI_2 were modulated differently. For example, in Fig 4A, ISI_1 was similar between the first (red) and second (green) events, but tended to be different for the third event (blue). In contrast, ISI_2 was similar between the secon[illegible]d thir[illegible]ents, but different for the first event. Accordingly, in the two-dimensional plot of ISI_1 and ISI_2, bursts of different events occupied different locations, and events did not align one-dimensionally, but were distributed two-di[illegible]

These pages are NOT the revised manuscript, but the original manuscript. They are provided to answer the reviewer's comments written in the PDF file of the original manuscript.

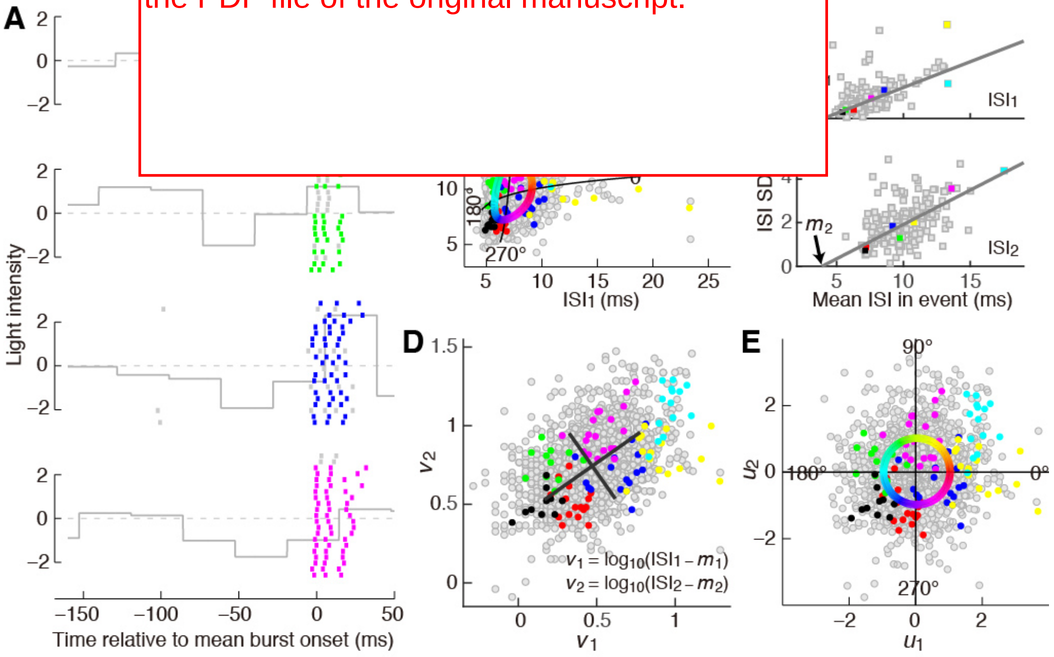

Fig 4. ISI patterns of three-spike bursts. Data from the cell shown in Fig 1. **(A)** Raster plot. Colored lines represent 3-spike bursts. Short gray lines are other spikes. The gray continuous line indicates the light intensity as shown in Fig 1A. **(B)** Distribution of ISI_1 and ISI_2. Each dot represents a 3-spike burst. Colored dots are bursts generated in seven different events, four of which are shown in (A) with the same color. Gray dots represent all other bursts. Black lines are the u_1 and u_2 axes. Burst phases are shown. The colors in the circle represent burst phases. **(C)** Variability of ISIs. Each dot represents an event. The horizontal and vertical axes are the average and SD of ISIs in an event, respectively. Gray lines show linear fits. **(D)** v_1 and v_2 were determined as indicated by the equations. Each dot represents a 3-spike burst. Black lines indicate the principle axes. The length of the lines represents the SD in the axis of the corresponding principle components. The coordinates of the crossing points are the averages of v_1 and v_2. **(E)** u_1 and u_2 determined by linear scaling of v_1 and v_2 along the principle axes. The colors in the circle show burst phases.

These pages are NOT the revised manuscript, but the original manuscript. They are provided to answer the reviewer's comments written in the PDF file of the original manuscript.

The above results [illegible] egrees of freedom. The distribution of IS[illegible] is. First, events with longer ISIs tended to [illegible] h shorter ISIs (Fig 4C). This inhomogene[illegible] ormation with a resolution higher than that represented by longer ISIs. Second, although ISI_1 and ISI_2 were modulated differently, they were not completely independent, but were correlated (Fig 4B; correlation coefficient = 0.16 ± 0.14, mean ± SD, n = 41). We were able to correct the first point by non-linearly scaling ISI_1 and ISI_2 so that different events had similar variability (Fig 4C and D). To correct the second point, variables were further linearly scaled to have an approximately circularly symmetric distribution so that the correlation was negligible (Fig 4E; correlation

coefficient = −0.01 ± 0.13, n = 41). The resultant variables, u_1 and u_2, had a circularly symmetric distribution and, therefore, were approximately independent, with different events occupying a similar amount of area (Fig 4E).

Using u_1 and u_2, we defined the "burst phase" for each burst (Fig 4B and E). Stimulus sequences preceding 3-spike bursts exhibited systematic differences according to the burst phase (Fig 5A). We subtracted the 3-BTA (Fig 5B) from the sequences preceding 3-spike bursts. The resulting deviations were oscillatory components with two or three ON peaks separated by 70–80 ms, with OFF peaks among them (Fig 5C–E). The intervals between these peaks were almost constant, but their timing relative to the onset of bursts shifted depending on the burst phase (Fig 5C–E). When the burst phase increased from 0° to 360°, the peaks moved closer to the burst onset (Fig 5C–E), and the timing of the major ON peaks showed approximately linear dependence on the bu [illegible] hase of 3-spike bursts encodes the temporal [illegible] und that the distance of the point (u_1, u_2) [illegible] litude of the oscillatory componen [illegible] d with natural scene movies (Fig 6).

These pages are NOT the revised manuscript, but the original manuscript. They are provided to answer the reviewer's comments written in the PDF file of the original manuscript.

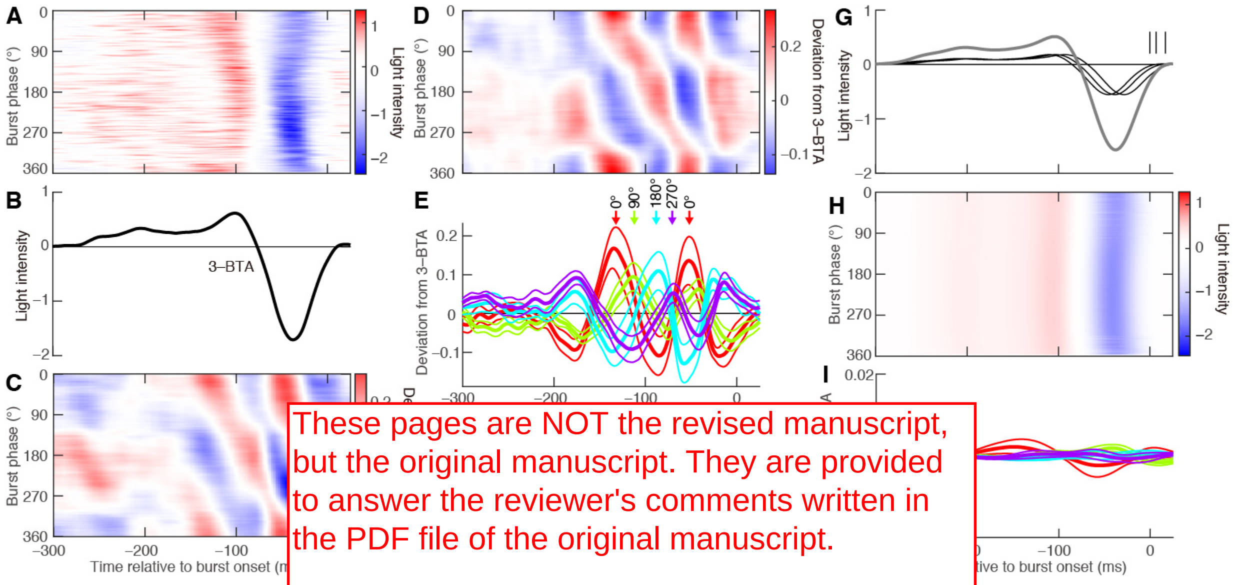

These pages are NOT the revised manuscript, but the original manuscript. They are provided to answer the reviewer's comments written in the PDF file of the original manuscript.

Fig 5. 3-spike burst IS[illegible]latory components. **(A–C)** Analysis of the cell shown in Fig 1. **(A)** Stimulus sequences preceding 3-spike bursts with different burst phases. **(B)** The average of all stimulus sequences preceding 3-spike bursts (3-BTA). **(C)** Data in (A) from which the 3-BTA is subtracted. **(D–F)** Population analyses. **(D)** Same analysis as in (C), averaged among 19 cells that generated at least 1200 3-spike bursts. **(E)** Thick lines indicate data in (D) at the indicated burst phases. Thin lines indicate the SEM values. Peaks around −100 ms are indicated. **(F)** Horizontal axis: $(u_1^2 + u_2^2)^{1/2}$, i.e., the distance of the point (u_1, u_2) from the origin in the u_1–u_2 plane in Fig 4E. Vertical axis: the root mean square of the oscillatory components between −200 and 25 ms. The error bars indicate SEM values for the 19 cells. **(G–I)** Reconstruction analyses. **(G)** Short vertical lines represent spikes in an example burst where ISI_1 = 7 ms and ISI_2 = 10 ms. Thin black lines indicate STAs calculated for 3-spike bursts of the cell used in (A–C), shifted according to the spikes in the example burst. Thick gray line indicates the reconstructed stimulus generated by adding the shifted STAs. **(H)** Analysis similar to (A) conducted for stimuli reconstructed as in (G) using the same bursts used

in (A). Color-coding is as used in (A). **(I)** Population analysis similar to (E), conducted for reconstructed stimuli.

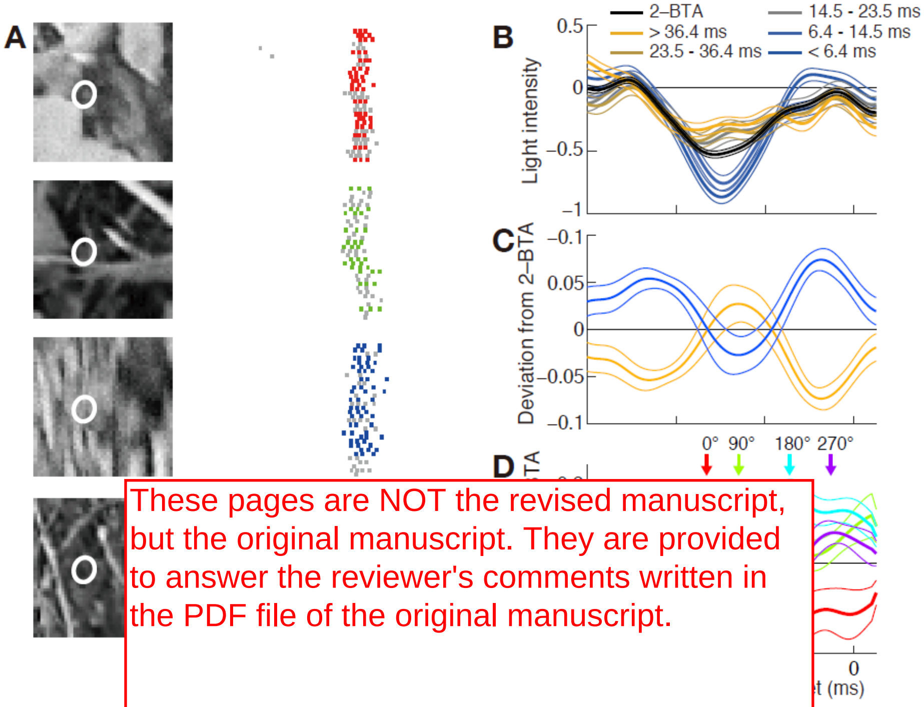

These pages are NOT the revised manuscript, but the original manuscript. They are provided to answer the reviewer's comments written in the PDF file of the original manuscript.

Fig 6. ISI analysis of [illegible] sponses of a single ganglion cell. (Right) Raster plots. The colored dots show 3-spike bursts. (Left) Image frames at −60 ms relative to the average timing of burst onsets. White ellipses show receptive field centers determined by reverse correlation and Gaussian fitting. Bar: 1 mm. **(B-D)** Analyses using light intensity at the receptive field center. **(B)** Analysis of 2-spike bursts of a single ganglion cell as shown in Fig 3E. Thin lines indicate SEM values. **(C)** Population analysis of 2-spike bursts similar to Fig 3G. Thick lines indicate the average of the stimulus sequence preceding 2-spike bursts with the longest (orange) and shortest (blue) 50% of intraburst ISIs, from which 2-BTA is subtracted. Thin lines indicate the SEM values. Data from 13 cells that generated more than 800 2-spike bursts. **(D)** Population analysis of 3-spike bursts similar to Fig 5E. Stimulus sequences

preceding 3-spike bursts with different phases. The 3-BTA is subtracted. The thick and thin lines indicate the average and SEM values among 8 cells that generated more than 1000 3-spike bursts.

As shown in Fig 4B, bursts with the phase 0° and 180° had only ~5- and ~1-ms differences in ISI_1 and ISI_2, respectively. Nevertheless, for bursts with the phase 0°, the ON and OFF peaks in the encoded sequences were separated by ~80 ms, while the separation was only ~50 ms for bursts with the phase 180° (Fig 5A). These results suggest that single spikes in bursts do not simply indicate the occurrence of a single characteristic in light intensity sequence. To further confirm this point, we conducted a simple reconstruction analysis. We calculated the spike-triggered averages (STA) for three-spike bursts and then generated an estimated stimulus sequence by adding the STA aligned according to the burst spikes (Fig 5G). This reconstruction failed to reproduce the observed dependence of the ON and OFF peaks on the burst phases (compare Fig 5A and H), and the deviation from the 3-BTA was much smaller than that of the actual data (compare [illegible]tion, the root mean square of the deviati[illegible]een −200 and +25 ms and averaged for all [illegible]he actual stimuli than for the reconstructed [illegible]0.002 for reconstructed stimuli, $n = 19$, $P = 1.5 \times 10^{-7}$, two-tailed Mann–Whitney–Wilcoxon test). Thus, the simple reconstruction model failed to explain the observed burst coding.

These pages are NOT the revised manuscript, but the original manuscript. They are provided to answer the reviewer's comments written in the PDF file of the original manuscript.

Two independent components of burst patterns

The oscillatory component encoded by 2-spike burst ISIs had peak-to-peak intervals that are similar to those of the components encoded by 3-spike bursts (~80 ms; compare Fig 3G and 5E),

suggesting that 2- and 3-spike burst ISIs are modulated by related stimulus features. To further characterize this similarity, we analyzed events in which both 2- and 3-spike bursts were generated (e.g., Fig 4A, bottom). For each of these events we calculated the average values of u_1 and u_2 for 3-spikes bursts and the average value of the 2-spike burst ISIs (Fig 7A). Plotting the data on the u_1-u_2 plane showed that 2-spike burst ISIs differ systematically depending on the position of the events along the direction of ~30° (Fig 7B). A linear fitting indicated that the optimum direction was 33.1° ± 15.6° (circular mean ± SD, *n* = 41; Fig 7C, magenta), suggesting that 2-spike burst ISIs are modulated by an oscillatory component that modulates 3-spike burst patterns in the orientation of ~33.1° on the u_1-u_2 plane.

These pages are NOT the revised manuscript, but the original manuscript. They are provided to answer the reviewer's comments written in the PDF file of the original manuscript.

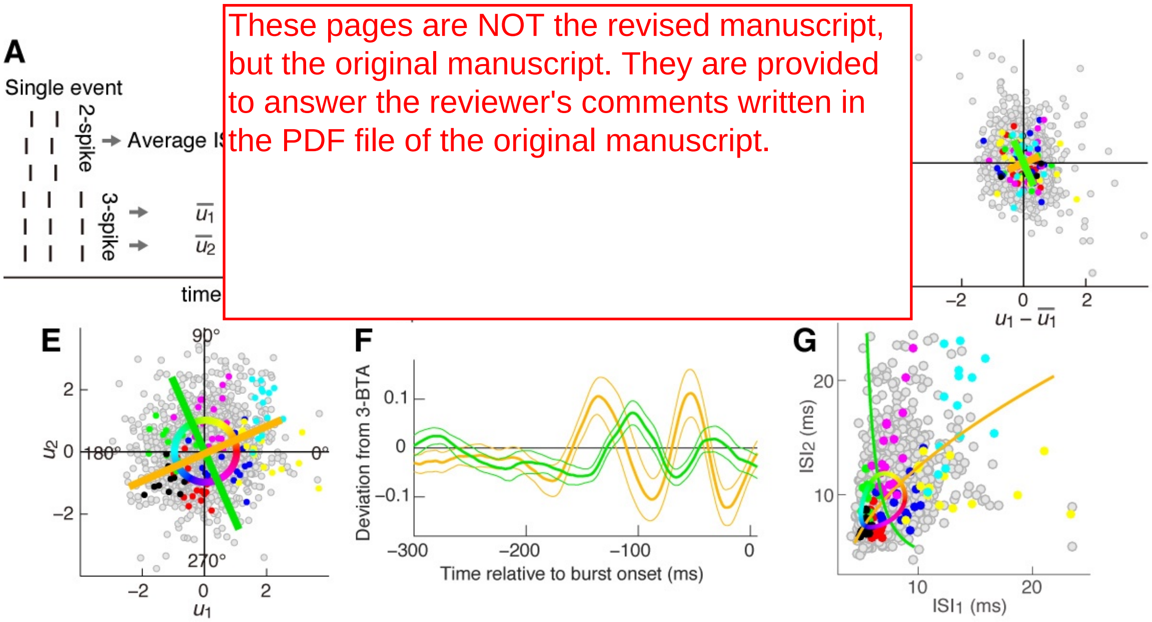

Fig 7. Identification of independent components that determine burst patterns. (A) The average value of 2-spike burst ISI and the average values of 3-spike burst u_1 and u_2 were determined for each event. **(B)** Dependence of 2-spike burst ISIs on 3-spike burst patterns. Each dot represents an event in which the cell generated both 2- and 3-spike bursts. The horizontal and vertical axes are the averages of u_1 and u_2 of 3-spike bursts in an event, respectively. The

color indicates the average ISI of 2-spike bursts. The arrow shows the orientation of the increase of 2-spike burst ISIs determined by linear fitting. **(C)** Population analyses for $n = 41$ cells. (Magenta) The orientation of the increase of 2-spike burst ISIs determined as in (B). (Orange and green) Principle axes of trial-to-trial variations of u_1 and u_2, determined as in (D). Orange shows the shorter axes. Dotted and thick lines are the circular histograms and average orientations, respectively. **(D–F)** Analyses of trial-to-trial variability. **(D)** Trial-to-trial variations of u_1 and u_2 in each event. Each dot represents a 3-spike burst. The orange and green lines indicate the orientation of the principle components through all events. The lengths represent the SD along the axes. Compare with Fig 4E. **(E)** The same panel as Fig 4E, shown with the orientations of the p[illegible] preceding bursts with the phases correspo[illegible] ta from 19 cells that generated at least 1[illegible] plotted on the ISI_1–ISI_2 plane.

These pages are NOT the revised manuscript, but the original manuscript. They are provided to answer the reviewer's comments written in the PDF file of the original manuscript.

The above result raises the hypothesis that the retinal mechanism that modulates 2-spike ISIs also modulates 3-spike burst patterns in the orientation of ~33.1° on the u_1-u_2 plane. Since u_1 and u_2 are suggested to have two degrees of freedom, one possibility is that another independent mechanism modulates 3-spike burst patterns in the orthogonal orientation, i.e., ~123.1°, on the u_1-u_2 plane. If two such independent mechanisms were present, modulation of u_1 and u_2 in the two orthogonal orientations would have independent trial-to-trial variations, and we tested this prediction. Although u_1 and u_2 had an approximately circularly symmetric distribution (Fig 4E), their trial-to-trial variations within each event had an asymmetric distribution (Fig 7D). Principal component analysis of this distribution indicated that the

principal axes corresponding to the smaller and larger variances were in the orientations of 29.4° ± 7.3° and 119.4° ± 7.3° (*n* = 41; orange and green in Fig 7C and D). This analysis indicates that modulations of u_1 and u_2 in these two orientations have approximately independent trial-to-trial variation. Because these axes (~29.4° and ~119.4°) are close to those proposed for the hypothesis (~33.1° and ~123.1°, see Fig 7C), the results are in accordance with the presence of two independent mechanisms, one of which (~30°) is common to 2- and 3-spike bursts. Consistently, the oscillatory component encoded by 3-spike bursts with the phase of the common component orientation (29.4° ± 7.3°) was similar to that encoded by 2-spike burst ISIs (compare Fig. 7F, yellow and Fig 3G, yellow). Given that the total distribution of u_1 and u_2 is circularly symmetric, the smaller trial-to-trial variability of the ~30° component as compared with the ~120° component ind[illegible]omponent common to 2- and 3-spike burs[illegible]sts.

These pages are NOT the revised manuscript, but the original manuscript. They are provided to answer the reviewer's comments written in the PDF file of the original manuscript.

The two approxi[illegible]mponents that are approximately orthog[illegible]ifference in phase (Fig 7F). This result suggests that the two orthogonal oscillatory stimulus components modulate 3-spike burst patterns in the orthogonal orientations on the u_1-u_2 plane. This model is consistent with the above result that 3-spike burst patterns encode the amplitude and phase of an oscillatory component, since an oscillatory sequence with an arbitrary amplitude and phase can be approximated by a sum of two orthogonal oscillatory components with fixed phases and the same frequency.

Discussion

Our results reveal that intraburst ISIs of retinal bursts encode oscillatory light intensity sequences that are much faster than the sequence encoded by the spike number. When a burst has three spikes, the two intraburst ISIs combinatorially encode the amplitude and phase of the oscillatory component. These results therefore suggest that a k-spike burst (k = 2, 3) encodes k different stimulus features by exploiting all the k degrees of freedom, i.e., the spike number and k−1 ISIs. This simultaneous representation of multiple stimulus features enables multiplexed information coding, a mechanism that greatly increases the information transmission capacity [19, 27, 28]. Whether this combinatorial coding occurs for bursts with $k \geq 4$ spikes remains unknown, as these bursts were rarel[illegible]).

These pages are NOT the revised manuscript, but the original manuscript. They are provided to answer the reviewer's comments written in the PDF file of the original manuscript.

Mechanisms of the co[illegible]

Figure 8 shows a codi[illegible]litude of slow light intensity modulation d[illegible]ISIs are regulated by two independent mechanisms that are driven by orthogonal fast oscillatory stimulus components, as suggested by the comparison between 2- and 3-spike bursts and the analysis of trial-to-trial variation. When a burst contains two spikes, the ISI is regulated by one of the two mechanisms, and thus 2-spike burst ISIs encode the amplitude of an oscillatory component of a fixed phase. When a burst has three spikes, the two mechanisms combinatorially determine the two ISIs. Because the two mechanisms are driven by the two orthogonal oscillatory components, the two ISIs of 3-spike bursts carry information about both the amplitude and phase of the oscillatory component. Modulation of the 3-spike ISI pattern by the common component is similar to modulation of the burst duration, i.e, $ISI_1 + ISI_2$ (see yellow in Fig 7G).

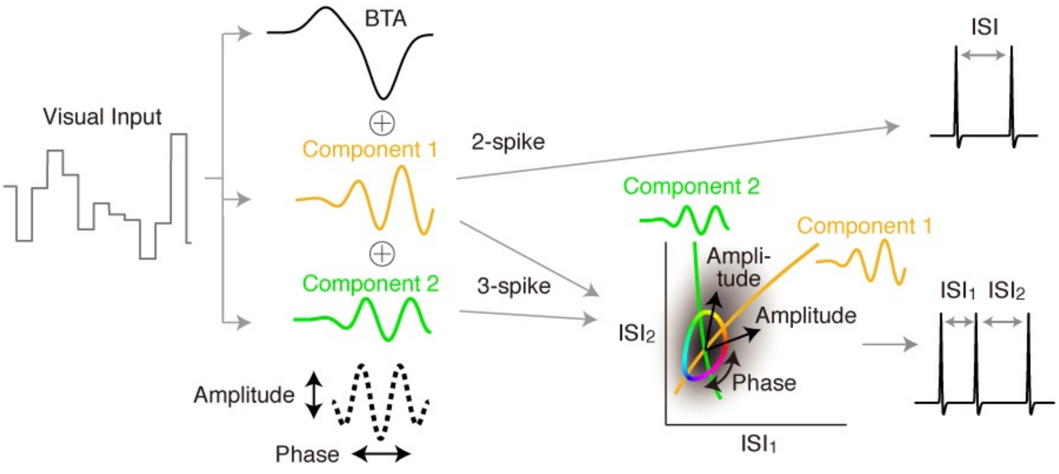

Fig 8. Schematic view of burst coding. The dotted line indicates the sum of the oscillatory components 1 and 2, whose amplitude and phase are encoded by the 3-spike burst pattern.

These pages are NOT the revised manuscript, but the original manuscript. They are provided to answer the reviewer's comments written in the PDF file of the original manuscript.

The two propose[illegible]oximately independent trial-to-trial variatio[illegible] on two largely non-overlapping synapti[illegible]nt temporal properties, considering that the two mechanisms respond to two different temporal sequences. In the vertebrate retinae, bipolar cells have ~10 subtypes [29-32], and different subtypes have distinct physiological properties [32-34] and varying temporal response characteristics [35-37]. In addition, the inhibitory effect of amacrine cells on bipolar cells generates further variation of temporal properties [38]. Therefore, specific subsets of bipolar and amacrine cells may constitute largely non-overlapping synaptic pathways underling the combinatorial coding.

Implications for visual processing

It is currently unclear to what extent the burst ISI information analyzed in this study is transmitted to the brain. However, in many brain regions, neuronal responses are sensitive to the

millisecond-scale temporal structure of synaptic inputs [24]. For example, in synaptic transmission from the retina to the lateral geniculate nucleus (LGN), retinal spikes with ISIs of a few milliseconds are much more effective in eliciting LGN spikes than those with ISIs of > ~20 ms [39-45]. Consistently, LGN burst ISIs are sensitive to the millisecond-scale structure of current input [19]. Similar to synaptic connections from the retina to the LGN, those from the LGN to cortical neurons are more responsive to spikes with short ISIs than those with long ISIs [46]. Such dependence of neuronal responses on input ISIs suggests that bursts with different ISIs elicit different spike responses of postsynaptic neurons. In addition, the dependence on ISIs varies among individ [illegible] ests that individual synapses have differe [illegible] ay function as a system to decode bur [illegible] ne dependence of ISIs on the temporal patte [illegible] patial patterns [47]. Therefore, it is possible that burst ISIs encode spatial information as well as temporal information.

These pages are NOT the revised manuscript, but the original manuscript. They are provided to answer the reviewer's comments written in the PDF file of the original manuscript.

Conclusions

The present results suggest that the retina employs mechanisms to regulate multiple components of intraburst ISIs, and thereby encodes multiple stimulus features by exploiting all degrees of freedom of burst spike patterns, i.e., the spike number and multiple intraburst ISIs. This burst coding is likely to affect visual information transmission, as synaptic transmission is sensitive to ISIs. Because bursts occur in various regions of the brain, analyses similar to the present study may reveal previously overlooked information transmission in those regions.

Materials and Methods

Animals

All experiments were approved by the RIKEN Wako animal experiments committee and were performed according to the guidelines of the animal facilities of the RIKEN Center for Brain Science. Larval tiger salamanders were provided by Charles D. Sullivan Co. Inc., Nashville, Tennessee, USA.

Recording and Stimulation

Retinal recording was performed as described previously [48]. Dark-adapted retinae from larval male and female tiger salamanders were isolated in oxygenated Ringer's medium at 25 °C. A piece of the retina (2– [illegible] croelectrodes (MED-P2H07A, Alpha MED [illegible] with oxygenated Ringer's solution (2 m [illegible] y refreshment at 30 Hz; mean and SD of t [illegible] as projected through an objective lens using a CRT monitor (60-Hz refresh rate; E551, Dell Inc., Round Rock, Texas, USA) controlled by the Matlab Psychophysics Toolbox [49, 50] or a light-emitting diode (E1L53-AWOC2-01 5-B5, Toyoda Gosei, Japan). The light intensity sequence was a random Gaussian sequence (65.5–183.3 s). The same sequence was repeated typically more than 20 times. For the natural scene stimulation, 200 s of a movie [51] was projected at 30 Hz using the CRT monitor (64 × 64 pixels, 60.6 μm/pixel; the mean intensity was 4.0 mW/m^2). Amplified voltage signals from the electrodes were stored and action potentials of single units were isolated using a Matlab program (a gift from Dr. Stephan A. Baccus). Analyses were performed using stable cells with mean firing rates >1.7 Hz.

These pages are NOT the revised manuscript, but the original manuscript. They are provided to answer the reviewer's comments written in the PDF file of the original manuscript.

Identification of Bursts and Events

Histograms of the ISIs were generated for each isolated ganglion cell. The histograms often had two distinct peaks (Fig 1B) representing shorter and longer ISIs, corresponding to intra- and interburst ISIs, respectively [3-5]. The threshold interval T_{thresh} was set at the trough between the two peaks in the ISI histogram (Fig 1B). T_{thresh} was 38.6 ± 20.0 ms (mean ± SD) for 41 cells stimulated with the spatially uniform stimulation, and 87.7 ± 18.5 ms for the 16 cells stimulated with the natural scene movie. If two consecutive spikes occurred with an interval shorter than T_{thresh}, they were incorporated into the same burst, while they were separated into two consecutive bursts if the interval was longer than T_{thresh} (Fig 1C). The robustness of this method was examined as follows. Bursts were defined using various threshold intervals of ~10 ms to ~100 ms, and the rates of the isolated spikes and b[illegible], and r_{+10} (Hz) denote the rates of the 2-spi[illegible], T_{thresh}, and T_{thresh}+10 ms, respectively. The [illegible] $/r_0$, was 0.021 ± 0.020 (mean ± SD, n = 41) [illegible]on, and 0.022 ± 0.014 for the 16 cells stimulated with the natural scene movie. These small values indicate the robustness of the method. The median intraburst ISIs of the 2-spike bursts and the median of the duration (ISI_1 + ISI_2) of the 3-spike bursts were 8.1 ± 2.8 and 14.4 ± 4.7 ms (mean ± SD, n = 41), respectively, for the cells stimulated with the spatially uniform stimulation, and 15.4 ± 5.6 and 29.0 ± 9.8 ms, respectively, for the 16 cells stimulated with the natural scene movie.

These pages are NOT the revised manuscript, but the original manuscript. They are provided to answer the reviewer's comments written in the PDF file of the original manuscript.

Events were determined as follows. The first spikes of bursts were extracted (Fig 1E, top) and merged across different repeats of the stimulus presentation (black in Fig 1E, middle). The intervals of these merged first spikes were then measured and a histogram was generated (Fig 1F). The histogram had two peaks separated by T_{thresh} (red and blue in Fig 1F), indicating that the intervals

were composed of short intervals representing inter-trial fluctuation (red in Fig 1E, middle, and red in Fig 1F) and longer intervals separating the consecutive events (blue in Fig 1E, middle, and blue in Fig 1F). Thus, if two consecutively merged first spikes were closer than T_{thresh}, they were incorporated into the same event; otherwise, they were assigned into two consecutive events (Fig 1E, bottom). The robustness of the method was evaluated as follows. If bursts occurred with a large timing jitter in different repeats, two consecutive bursts in one repeat were incorporated into one event. However, this occurred for only 2.7% ± 2.7% of bursts for the cells (mean ± SD, $n = 41$) stimulated with the spatially uniform stimulation, and 3.5% ± 1.1% for the cells stimulated with the natural scene movie (n [illegible]ion of events was robust.

These pages are NOT the revised manuscript, but the original manuscript. They are provided to answer the reviewer's comments written in the PDF file of the original manuscript.

Experimental Desig[illegible]

The numbers of the a[illegible]s: $n = 41$ cells in 15 retinae for spatially uniform stimulation, and $n = 16$ cells in 4 retinae for natural scene stimulation.

Correlations between the spike number and the intraburst ISIs of the bursts were investigated using data from ganglion cells stimulated with the spatially uniform stimulation as follows. For each event j in which at least one 2-spike burst occurred, the average number of spikes ($n^{(j)}$) and the average intraburst ISIs of the 2-spike bursts ($m^{(j)}$) were determined. The correlation between $n^{(j)}$ and $m^{(j)}$ was then calculated across events. Similar correlations were calculated for ISI_1 and ISI_2 of the 3-spike bursts.

Mutual information conveyed by the burst spike number was determined as follows. When the stimulation was repeated, bursts occurred in a limited number of discrete events that were defined at specific time points of the sequence (Fig 1A). Therefore, it was investigated how far

the receiver of bursts can specify the events by knowing the spike number, as compared to the case where the receiver receives bursts without knowing the spike number. N_{Rep} and N_{Ev} represent the number of stimulus repeats and the number of events, respectively. $n_k^{(j)}$ represents the number of k-spike bursts that occurred in the j-th event during the N_{Rep} repeats of stimulation $(k = 0,1,2, \cdots, k_{\max}; j = 1, \cdots, N_{\mathrm{Ev}})$, where $k_{\max}$ is the largest number of spikes in a burst. For all j, $\sum_{k=0}^{k_{\max}} n_k^{(j)} = N_{\mathrm{Rep}}$. The number of all bursts that occurred in the j-th event is $n_{\mathrm{Burst}}^{(j)} = \sum_{k=1}^{k_{\max}} n_k^{(j)}$, and the total number of bursts is $N_{\mathrm{Burst}} = \sum_{j=1}^{N_{\mathrm{Ev}}} n_{\mathrm{Burst}}^{(j)}$. When a burst was generated, the pro[illegible] N_{Burst}. Thus, the prior entropy of events, calc[illegible] without knowing the spike number, is H_{Burs}[illegible] posterior probability that the burst was in th[illegible] total number of k-spike bursts. Thus, the posterior entropy is $H_{k\text{-spike}} = \sum_{j=1}^{N_{\mathrm{Ev}}} \frac{n_k^{(j)}}{N_k} \log_2 \frac{n_k^{(j)}}{N_k}$. The mutual information is $I_{\mathrm{number}} = H_{\mathrm{Burst}} - \sum_{k=1}^{k_{\max}} p(k\text{-spike}) \cdot H_{k\text{-spike}}$, where $p(k\text{-spike}) = N_k / N_{\mathrm{Burst}}$ is the probability of k-spike bursts. To evaluate the statistical significance, surrogate data were generated by exchanging the spike number of each burst with that of a randomly selected burst. One hundred surrogate data were generated and $I_{\mathrm{number}}^{\mathrm{sur}\,l}$ was calculated for the l-th surrogate $(l = 1, \cdots, 100)$. If N_{larger} of $I_{\mathrm{number}}^{\mathrm{sur}\,l}$ was larger than I_{number}, the statistical significance is $P = N_{\mathrm{larger}}/100$. The estimated mutual information was corrected by the bias correction method [52].

These pages are NOT the revised manuscript, but the original manuscript. They are provided to answer the reviewer's comments written in the PDF file of the original manuscript.

Information conveyed by intraburst ISIs was calculated as follows. Intraburst ISIs of 2-spike bursts, and ISI_1 and ISI_2 of 3-spike bursts were divided into 4 groups according to the length of the ISIs, so that each group had as equal number of ISIs as much as possible. Two- and 3-spike

bursts were thus divided into 4 and 16 groups, respectively. $n_{k,q}^{(j)}$ denotes the number of k-spike bursts of the group q in the j-th event (q = 1, ⋯, $q_{\max}$; $q_{\max}$ = 4 for k = 2, $q_{\max}$ = 16 for k = 3). The total number of k-spike bursts of the group q is $N_{k,q} = \sum_{j=1}^{N_{\mathrm{Ev}}} n_{k,q}^{(j)}$. When a k-spike burst of the group p occurred, the posterior entropy is $H_{k,q} = \sum_{j=1}^{N_{\mathrm{Ev}}} \frac{n_{k,q}^{(j)}}{N_{k,q}} \log_2 \frac{n_{k,q}^{(j)}}{N_{k,q}}$. The information conveyed by ISIs is $I_{k\text{-spike ISI}} = H_{k\text{-spike}} - \sum_{q=1}^{q_{\max}} p(\text{group } q|k\text{-spike}) \cdot H_{k,q}$, where $p(\text{group } q|k\text{-spike}) = N_{k,q}/N_k$. Statistical significance was evaluated similar to the spike number analysis. The information va [illegible]

These pages are NOT the revised manuscript, but the original manuscript. They are provided to answer the reviewer's comments written in the PDF file of the original manuscript.

Coordinate [illegible]s follows (Fig 4B–D). For 3-spike bursts in [illegible]re designated as $\overline{\mathrm{ISI}}_1^{(j)}$ and $\mathrm{SD}_1^{(j)}$, respecti[illegible] , N_{Ev}) as $\mathrm{SD}_1^{(j)} \cong a_1 \left(\overline{\mathrm{ISI}}_1^{(j)} - m_1\right)$, where a_1 and m_1 are constants (Fig 4C, top). For a variable $v_1 = \log_{10}(\mathrm{ISI}_1[\mathrm{ms}] - m_1[\mathrm{ms}])$, $\frac{dv_1}{d\mathrm{ISI}_1} = (\mathrm{ISI}_1 - m_1)^{-1}(\log 10)^{-1}$, and thus the standard deviation of v_1 in each event was similar among different events (see bursts shown in different colors in Fig 4D). Bursts with $\mathrm{ISI}_1 \leq m_1$ were removed from the analysis. v_2 was similarly defined for ISI_2 (Fig 4C, bottom). The principle axes of the distribution of v_1 and v_2 were determined (Fig 4D), and new variables u_1 and u_2 were defined by scaling v_1 and v_2 along these axes so that the standard deviations along these axes were equal to 1 (Fig 4E). u_1 and u_2 were shifted so that their averages were zero. The burst phase was $\mathrm{atan2}(u_2, u_1)$ (Fig 4E).

To characterize the stimulus features encoded by bursts, the stimulus sequences preceding bursts of a specific spike number, intraburst ISIs within a specific range, or burst phase within a specific range, were collected. The average and SEM values of the collected sequences were then

used for the analyses. Neurons that generated only small numbers of 2- or 3-spike bursts were removed from the analyses (see legends for Figs 3G, 5D, 6C, and 6D). For linear reconstruction, stimulus sequences preceding the spikes in three-spike bursts were collected and averaged. The average sequence was divided by three and then used as the STA for the reconstruction (Fig 5G–I).

Data Availability

The central data and computer codes used in this paper are available the open science framework database at: https://osf.io/29ect/. Other data and codes are available upon request.

Acknowledgeme[illegible]

These pages are NOT the revised manuscript, but the original manuscript. They are provided to answer the reviewer's comments written in the PDF file of the original manuscript.

We thank Dr. Markus [illegible] unstinting encouragement. We a[illegible] nagihara, Neal Hessler, Yoshihiro Yo[illegible] Lesica, Xin Jing, Charles Yokoyama, Naoki Masuda, Shiro Ikeda, Makoto Kaneda, Tomoki Fukai, Keita Watanabe, and Tetsuya Haga for thoughtful discussions.

Author contributions

Conceptualization: Toshihiko Hosoya.

Data curation: Toshiyuki Ishii, Toshihiko Hosoya.

Formal analysis: Toshiyuki Ishii, Toshihiko Hosoya.

Funding acquisition: Toshihiko Hosoya.

Investigation: Toshiyuki Ishii.

Methodology: Toshiyuki Ishii, Toshihiko Hosoya.

Project administration: Toshihiko Hosoya.

Resources: Toshihiko Hosoya.

Software: Toshihiko Hosoya.

Supervision: Toshihiko Hosoya.

Validation: Toshiyuki Ishii, Toshihiko Hosoya.

Visualization: Toshiyuki Ishii, Toshihiko Hosoya.

Writing – original draft: Toshihiko Hosoya.

Writing – review & editing: Toshiyuki Ishii, Toshihiko Hosoya.

These pages are NOT the revised manuscript, but the original manuscript. They are provided to answer the reviewer's comments written in the PDF file of the original manuscript.

References

1. Brenner N, Strong [illegible] RR. Synergy in a neural code. Neur[illegible] 35917.
2. Rieke FDW, de Ruyter van Steveninck R, Bialek W. Spikes: Cambridge, Massashusetts: MIT Press; 1997.
3. Berry MJ, Warland DK, Meister M. The structure and precision of retinal spike trains. Proc Natl Acad Sci U S A. 1997;94(10):5411-6. doi: 10.1073/pnas.94.10.5411. PubMed PMID: 9144251; PubMed Central PMCID: PMCPMC24692.
4. Keat J, Reinagel P, Reid RC, Meister M. Predicting every spike: a model for the responses of visual neurons. Neuron. 2001;30(3):803-17. doi: 10.1016/s0896-6273(01)00322-1. PubMed PMID: 11430813.
5. Reinagel P, Godwin D, Sherman SM, Koch C. Encoding of visual information by LGN bursts. J Neurophysiol. 1999;81(5):2558-69. doi: 10.1152/jn.1999.81.5.2558. PubMed

PMID: 10322089.

6. Denning KS, Reinagel P. Visual control of burst priming in the anesthetized lateral geniculate nucleus. J Neurosci. 2005;25(14):3531-8. doi: 10.1523/JNEUROSCI.4417-04.2005. PubMed PMID: 15814783; PubMed Central PMCID: PMCPMC6725375.
7. Lisman JE. Bursts as a unit of neural information: making unreliable synapses reliable. Trends Neurosci. 1997;20(1):38-43. doi: 10.1016/S0166-2236(96)10070-9. PubMed PMID: 9004418.
8. Oswald AM, Chacron MJ, Doiron B, Bastian J, Maler L. Parallel processing of sensory input by bursts and isolated spikes. J Neurosci. 2004;24(18):4351-62. doi: 10.1523/JNEUR[illegible] Central PMCID: PMCPMC67294[illegible]
9. Krahe R, Gabbia[illegible]i. 2004;5(1):13-23. doi: 10.1038/nrn[illegible]
10. Zeldenrust F, Wadman WJ, Englitz B. Neural Coding With Bursts-Current State and Future Perspectives. Front Comput Neurosci. 2018;12:48. doi: 10.3389/fncom.2018.00048. PubMed PMID: 30034330; PubMed Central PMCID: PMCPMC6043860.
11. DeBusk BC, DeBruyn EJ, Snider RK, Kabara JF, Bonds AB. Stimulus-dependent modulation of spike burst length in cat striate cortical cells. J Neurophysiol. 1997;78(1):199-213. doi: 10.1152/jn.1997.78.1.199. PubMed PMID: 9242274.
12. Eggermont JJ, Smith GM. Burst-firing sharpens frequency-tuning in primary auditory cortex. Neuroreport. 1996;7(3):753-7. doi: 10.1097/00001756-199602290-00018. PubMed PMID: 8733738.
13. Eyherabide HG, Rokem A, Herz AV, Samengo I. Burst firing is a neural code in an insect

These pages are NOT the revised manuscript, but the original manuscript. They are provided to answer the reviewer's comments written in the PDF file of the original manuscript.

auditory system. Front Comput Neurosci. 2008;2:3. doi: 10.3389/neuro.10.003.2008. PubMed PMID: 18946533; PubMed Central PMCID: PMCPMC2525941.

14. Eyherabide HG, Rokem A, Herz AV, Samengo I. Bursts generate a non-reducible spike-pattern code. Front Neurosci. 2009;3(1):8-14. doi: 10.3389/neuro.01.002.2009. PubMed PMID: 19753092; PubMed Central PMCID: PMCPMC2695386.

15. Marsat G, Pollack GS. The structure and size of sensory bursts encode stimulus information but only size affects behavior. J Comp Physiol A Neuroethol Sens Neural Behav Physiol. 2010;196(4):315-20. doi: 10.1007/s00359-010-0514-8. PubMed PMID: 20213110.

16. Marsat G, Pollack GS. Bursting neurons and ultrasound avoidance in crickets. Front Neurosci. 2012;6[illegible]2783158; PubMed Central PMCID: [illegible]

17. Martinez-Conde S[illegible]pikes during visual fixation in the aw[illegible]ual cortex. Proc Natl Acad Sci U S A. 2002;99(21):13920-5. doi: 10.1073/pnas.212500599. PubMed PMID: 12361982; PubMed Central PMCID: PMCPMC129798.

These pages are NOT the revised manuscript, but the original manuscript. They are provided to answer the reviewer's comments written in the PDF file of the original manuscript.

18. Mathy A, Ho SS, Davie JT, Duguid IC, Clark BA, Hausser M. Encoding of oscillations by axonal bursts in inferior olive neurons. Neuron. 2009;62(3):388-99. doi: 10.1016/j.neuron.2009.03.023. PubMed PMID: 19447094; PubMed Central PMCID: PMCPMC2777250.

19. Mease RA, Kuner T, Fairhall AL, Groh A. Multiplexed Spike Coding and Adaptation in the Thalamus. Cell Rep. 2017;19(6):1130-40. doi: 10.1016/j.celrep.2017.04.050. PubMed PMID: 28494863; PubMed Central PMCID: PMCPMC5554799.

20. Alitto HJ, Weyand TG, Usrey WM. Distinct properties of stimulus-evoked bursts in the

lateral geniculate nucleus. J Neurosci. 2005;25(2):514-23. doi: 10.1523/JNEUROSCI.3369-04.2005. PubMed PMID: 15647497; PubMed Central PMCID: PMCPMC6725468.

21. Lesica NA, Stanley GB. Encoding of natural scene movies by tonic and burst spikes in the lateral geniculate nucleus. J Neurosci. 2004;24(47):10731-40. doi: 10.1523/JNEUROSCI.3059-04.2004. PubMed PMID: 15564591; PubMed Central PMCID: PMCPMC6730113.

22. Lesica NA, Weng C, Jin J, Yeh CI, Alonso JM, Stanley GB. Dynamic encoding of natural luminance sequences by LGN bursts. PLoS Biol. 2006;4(7):e209. doi: 10.1371/journal.pbio.0040209. PubMed PMID: 16756389; PubMed Central PMCID: PMCPMC14757[illegible]

23. Butts DA, Desbo[illegible]pisodic nature of spike trains in the[illegible]371-87. doi: 10.1152/jn.0007[illegible]MCID: PMCPMC3007659.

These pages are NOT the revised manuscript, but the original manuscript. They are provided to answer the reviewer's comments written in the PDF file of the original manuscript.

24. Izhikevich EM, Desai NS, Walcott EC, Hoppensteadt FC. Bursts as a unit of neural information: selective communication via resonance. Trends Neurosci. 2003;26(3):161-7. doi: 10.1016/S0166-2236(03)00034-1. PubMed PMID: 12591219.

25. Oswald AM, Doiron B, Maler L. Interval coding. I. Burst interspike intervals as indicators of stimulus intensity. J Neurophysiol. 2007;97(4):2731-43. doi: 10.1152/jn.00987.2006. PubMed PMID: 17409176.

26. Gollisch T, Meister M. Rapid neural coding in the retina with relative spike latencies. Science. 2008;319(5866):1108-11. doi: 10.1126/science.1149639. PubMed PMID: 18292344.

27. Fairhall AL, Lewen GD, Bialek W, de Ruyter Van Steveninck RR. Efficiency and ambiguity in an adaptive neural code. Nature. 2001;412(6849):787-92. doi: 10.1038/35090500. PubMed PMID: 11518957.

28. Panzeri S, Brunel N, Logothetis NK, Kayser C. Sensory neural codes using multiplexed temporal scales. Trends Neurosci. 2010;33(3):111-20. Epub 2010/01/05. doi: 10.1016/j.tins.2009.12.001. PubMed PMID: 20045201.

29. Ghosh KK, Bujan S, Haverkamp S, Feigenspan A, Wassle H. Types of bipolar cells in the mouse retina. J Comp Neurol. 2004;469(1):70-82. doi: 10.1002/cne.10985. PubMed PMID: 14689473.

These pages are NOT the revised manuscript, but the original manuscript. They are provided to answer the reviewer's comments written in the PDF file of the original manuscript.

30. MacNeil MA, H[illegible]opulation of bipolar cells in the rabbi[illegible]002/cne.20063. PubMed PMID: [illegible]

31. Pignatelli V, Stre[illegible]orphological study. J Comp Neurol. 2004;476(3):254-66. doi: 10.1002/cne.20207. PubMed PMID: 15269969.

32. Wu SM, Gao F, Maple BR. Functional architecture of synapses in the inner retina: segregation of visual signals by stratification of bipolar cell axon terminals. J Neurosci. 2000;20(12):4462-70. PubMed PMID: 10844015; PubMed Central PMCID: PMCPMC6772452.

33. Awatramani GB, Slaughter MM. Origin of transient and sustained responses in ganglion cells of the retina. J Neurosci. 2000;20(18):7087-95. PubMed PMID: 10995856; PubMed Central PMCID: PMCPMC6772807.

34. DeVries SH. Bipolar cells use kainate and AMPA receptors to filter visual information into separate channels. Neuron. 2000;28(3):847-56. doi: 10.1016/s0896-6273(00)00158-6.

PubMed PMID: 11163271.

35. Euler T, Haverkamp S, Schubert T, Baden T. Retinal bipolar cells: elementary building blocks of vision. Nat Rev Neurosci. 2014;15(8):507-19. PubMed PMID: 25158357.

36. Ichinose T, Fyk-Kolodziej B, Cohn J. Roles of ON cone bipolar cell subtypes in temporal coding in the mouse retina. J Neurosci. 2014;34(26):8761-71. doi: 10.1523/JNEUROSCI.3965-13.2014. PubMed PMID: 24966376; PubMed Central PMCID: PMCPMC4069354.

37. Ichinose T, Hellmer CB. Differential signalling and glutamate receptor compositions in the OFF bipolar cell t[illegible]94. doi: 10.1113/JP27145[illegible]D: PMCPMC47532[illegible]

These pages are NOT the revised manuscript, but the original manuscript. They are provided to answer the reviewer's comments written in the PDF file of the original manuscript.

38. Franke K, Berens [illegible] decorrelates visual feature representa[illegible]44. doi: 10.1038/nature21394. PubMed PMID: 28178238; PubMed Central PMCID: PMCPMC5325673.

39. Levine MW, Cleland BG. An analysis of the effect of retinal ganglion cell impulses upon the firing probability of neurons in the dorsal lateral geniculate nucleus of the cat. Brain Res. 2001;902(2):244-54. doi: 10.1016/s0006-8993(01)02411-8. PubMed PMID: 11384618.

40. Mastronarde DN. Two classes of single-input X-cells in cat lateral geniculate nucleus. II. Retinal inputs and the generation of receptive-field properties. J Neurophysiol. 1987;57(2):381-413. doi: 10.1152/jn.1987.57.2.381. PubMed PMID: 3559685.

41. Rathbun DL, Warland DK, Usrey WM. Spike timing and information transmission at retinogeniculate synapses. J Neurosci. 2010;30(41):13558-66. doi:

10.1523/JNEUROSCI.0909-10.2010. PubMed PMID: 20943897; PubMed Central PMCID: PMCPMC2970570.

42. Rowe MH, Fischer Q. Dynamic properties of retino-geniculate synapses in the cat. Vis Neurosci. 2001;18(2):219-31. doi: 10.1017/s0952523801182076. PubMed PMID: 11417797.

43. Sincich LC, Adams DL, Economides JR, Horton JC. Transmission of spike trains at the retinogeniculate synapse. J Neurosci. 2007;27(10):2683-92. doi: 10.1523/JNEUROSCI.5077-06.2007. PubMed PMID: 17344406; PubMed Central PMCID: PMCPMC6672514.

These pages are NOT the revised manuscript, but the original manuscript. They are provided to answer the reviewer's comments written in the PDF file of the original manuscript.

44. Usrey WM, Repp[illegible] efficacy of retinal inputs to the thala[illegible]487. PubMed PMID: 9759728.

45. Weyand TG. Reti[illegible]siol. 2007;98(2):769-85. doi: 10.1152/jn.00929.2006. PubMed PMID: 17553944.

46. Usrey WM, Alonso JM, Reid RC. Synaptic interactions between thalamic inputs to simple cells in cat visual cortex. J Neurosci. 2000;20(14):5461-7. PubMed PMID: 10884329.

47. Rathbun DL, Alitto HJ, Weyand TG, Usrey WM. Interspike interval analysis of retinal ganglion cell receptive fields. J Neurophysiol. 2007;98(2):911-9. doi: 10.1152/jn.00802.2006. PubMed PMID: 17522169; PubMed Central PMCID: PMCPMC2752417.

48. Meister M, Pine J, Baylor DA. Multi-neuronal signals from the retina: acquisition and analysis. J Neurosci Methods. 1994;51(1):95-106. doi: 10.1016/0165-0270(94)90030-2. PubMed PMID: 8189755.

49. Brainard DH. The Psychophysics Toolbox. Spat Vis. 1997;10(4):433-6. PubMed PMID: 9176952.

50. Pelli DG. The VideoToolbox software for visual psychophysics: transforming numbers into movies. Spat Vis. 1997;10(4):437-42. PubMed PMID: 9176953.

51. van Hateren JH. Processing of natural time series of intensities by the visual system of the blowfly. Vision Res. 1997;37(23):3407-16. doi: 10.1016/s0042-6989(97)00105-3. PubMed PMID: 9425553.

52. Panzeri S, Senatore R, Montemurro MA, Petersen RS. Correcting for the sampling bias problem in spike train information measures. J Neurophysiol. 2007;98(3):1064-72. doi: 10.1152/jn.00559.2007. PubMed PMID: 17615128.

These pages are NOT the revised manuscript, but the original manuscript. They are provided to answer the reviewer's comments written in the PDF file of the original manuscript.
