# Supplementary material for: Interspike intervals within retinal spike bursts combinatorially encode multiple stimulus features
Source: PLoS Comput Biol. 2020 Nov 6;16(11):e1007726. doi: 10.1371/journal.pcbi.1007726 (PMC7738174; doi:10.1371/journal.pcbi.1007726)
Supplement: Attachment — Submitted filename: review_plos_2020_2.pdf [file pcbi.1007726.s008.pdf]

The manuscript has improved a lot. I appreciate the new mutual information calculation, the improved mathematical notation, the documentation with the data and code and the inclusion of projection values and also the calculation of the effects of alignment. My only point, except for a few minor text edits, is that for the mutual information calculation no bias correction is performed, see

- Treves, A., & Panzeri, S. (1995). The Upward Bias in Measures of Information Derived from Limited Data Samples. *Neural Computation*, *7*(2), 399–407. https://doi.org/10.1162/neco.1995.7.2.399
- Panzeri, S., & Treves, A. (1996). Analytical estimates of limited sampling biases in different information measures. *Network: Computation in Neural Systems*, *7*(1), 87–107. https://doi.org/10.1080/0954898X.1996.11978656
- Panzeri, S., Senatore, R., Montemurro, M. A., & Petersen, R. S. (2007). Correcting for the sampling bias problem in spike train information measures. *Journal of Neurophysiology*, *98*(3), 1064–1072. https://doi.org/10.1152/jn.00559.2007

Minor text edits:

- In appreciate the inclusion of the distributions of projection values. Differently from the 'standard' approach, the authors choose to project onto the difference instead of the BTA itself. While this is no problem, I would expect a few lines (2-3 sentences) in the main text about why the authors chose to do this.
- Moreover, the authors write on several occasions that they "projected the stimulus onto (…) and collected the values (…), then normalized the values to have a mean = 0 and an SD =1". I think that if you would do that, then all distributions would be standard Gaussians with mean=0 and SD=1, which is clearly not the case (fig 2C-D, 3I-J, 7H-K). So I think the authors mean something else, but I'm not quite sure what?
- The unnumbered main text equation between equations 1 and 2: shouldn't v_1 be v_d?
- In figure 2, I am not sure what the difference between C and D is. Could this be clarified?
- Fig 5 G-I is only treated after fig 6 in the main text, this is a bit confusing.
- On p. 10 is states 'litter' instead of 'little'.

Given that the authors perform a bias correction (or that they explain why they would not), I would recommend it for publications after a few minor text edits.
