# Supplementary material for: Interspike intervals within retinal spike bursts combinatorially encode multiple stimulus features
Source: PLoS Comput Biol. 2020 Nov 6;16(11):e1007726. doi: 10.1371/journal.pcbi.1007726 (PMC7738174; doi:10.1371/journal.pcbi.1007726)
Supplement: Attachment — Submitted filename: Letter200917.pdf [file pcbi.1007726.s009.pdf]

Response to Reviewer 3

The manuscript has improved a lot. I appreciate the new mutual information calculation, the improved mathematical notation, the documentation with the data and code and the inclusion of projection values and also the calculation of the effects of alignment. My only point, except for a few minor text edits, is that for the mutual information calculation no bias correction is performed, see

o Treves, A., & Panzeri, S. (1995). The Upward Bias in Measures of Information Derived from Limited Data Samples. Neural Computation, 7(2), 399–407. https://doi.org/10.1162/neco.1995.7.2.399

o Panzeri, S., & Treves, A. (1996). Analytical estimates of limited sampling biases in different information measures. Network: Computation in Neural Systems, 7(1), 87–107. https://doi.org/10.1080/0954898X.1996.11978656

o Panzeri, S., Senatore, R., Montemurro, M. A., & Petersen, R. S. (2007). Correcting for the sampling bias problem in spike train information measures. Journal of Neurophysiology, 98(3), 1064–1072. https://doi.org/10.1152/jn.00559.2007

We thank the reviewer for the comment on the issue of bias correction. We have tested the bias correction procedure that is composed of two steps: a quadratic extrapolation of information values and a bootstrap shuffling procedure (Phase-of-Firing Coding of Natural Visual Stimuli in Primary Visual Cortex, Current Biology 18, 375–380, 2008). Unfortunately, in our hand, the results heavily depended on the details of the analysis (e.g. the bin size) in a complicated manner. It was thus difficult to obtain a good estimate of the corrected values. Therefore, we are afraid that incorporation of the correction procedure could complicate the interpretation of the data.

On the other hand, the information conveyed by the spike number or ISIs was defined by calculating the difference of two information values (e.g. $I_{\text{number}} = I_{\text{burst, number}} - I_{\text{burst}}$). Given that information biases are positive, this subtraction should partially cancel the biases.

We thus prefer to report the result as it is without incorporating correction procedures.

Minor text edits:

- *In appreciate the inclusion of the distributions of projection values. Differently from the 'standard' approach, the authors choose to project onto the difference instead of the BTA itself. While this is no problem, I would expect a few lines (2-3 sentences) in the main text about why the authors chose to do this.*

We thank the reviewer for the suggestion. We have briefly explained the rationale for the BTA calculation at the beginning of the last paragraph in the section "Burst spike numbers encode the amplitude of light intensity modulation."

- *Moreover, the authors write on several occasions that they "projected the stimulus onto (...) and collected the values (...), then normalized the values to have a mean = 0 and an SD =1". I think that if you would do that, then all distributions would be standard Gaussians with mean=0 and SD=1, which is clearly not the case (fig 2C-D, 3I-J, 7H-K). So I think the authors mean something else, but I'm not quite sure what?*

We apologize for the insufficient description of the projection. Fig 2C was generated as follows. First, $s^{*}_{3-1,k}(t)$, the raw projected value for cell *k*, was normalized to define $s_{3-1,k}(t)$ (mean=0 and SD=1). The probability density distribution $P\left(s_{3-1,k}(t)\right)$ was averaged across all cell *k* to define $P(s_{3-1}(t))$, which is shown as a dashed line in Fig 2C. Note that $P(s_{3-1}(t))$ is very similar to a Gaussian with mean=0 and SD=1.

Next, $s_{3-1,k}(t)$ values at isolated spikes $\left(t_1^{(1)}, t_2^{(1)}, \cdots, t_{N_1}^{(1)}\right)$, i.e., $s_{3-1,k}\left(t_1^{(1)}\right), s_{3-1,k}\left(t_2^{(1)}\right), \cdots, s_{3-1,k}\left(t_{N_1}^{(1)}\right)$, were collected and their probability density distribution $P(s_{3-1,k}|\text{1-spike})$ was calculated. Because $t_1^{(1)}, t_2^{(1)}, \cdots, t_{N_1}^{(1)}$ were determined by the stimulus-response property of the retina, $s_{3-1,k}\left(t_1^{(1)}\right), s_{3-1,k}\left(t_2^{(1)}\right), \cdots, s_{3-1,k}\left(t_{N_1}^{(1)}\right)$ are generally non-ramdomly sampled from $P\left(s_{3-1,k}(t)\right)$, and thus $P(s_{3-1,k}|\text{1-spike})$ is generally not the Gaussian with mean=0 and SD=1. Consistently, the thickest line in Fig 2C showing $P(s_{3-1}|\text{1-spike})$, i.e., $P(s_{3-1,k}|\text{1-spike})$ averaged across all cell *k*, is different from the Gaussian with mean=0 and SD=1. Similarly, $P(s_{3-1}|\text{2-spike})$, $P(s_{3-1}|\text{3-spike})$, and

$P(s_{3-1}|\text{4-spike})$ shown in Fig 2C are different from the Gaussian with mean=0 and SD=1.

Figs 2D, 3I-J, and 7H-K similarly contain probability density distributions that are different from the Gaussian with mean=0 and SD=1. To clarify these points, we have revised the Materials and Methods ("Projection onto 3-BTA−1-BTA,""Deviation from 2-BTA,""Approximately independent components").

The unnumbered main text equation between equations 1 and 2: shouldn't v1 be vd?

We thank the reviewer for pointing out the typo. We have corrected it.

In figure 2, I am not sure what the difference between C and D is. Could this be clarified?

Fig 2C compares the projected values sampled at all stimulus time points with those sampled at isolated spikes, 2-spike bursts, 3-spike bursts, and 4-spike bursts. In contrast, Fig 2D compares the projected values sampled at all isolated spikes and bursts with those sampled at isolated spikes, 2-spike bursts, 3-spike bursts, and 4-spike bursts. For this purpose, for Fig. 2C the projected values were collected at all stimulus time points and normalized so that mean=0 and SD=1 (dashed line in Fig 2C). In Fig 2D, the projected values were collected at isolated spikes and bursts and then normalized (dashed line in Fig 2D). We have described the rationale in the beginning of the last paragraph of the section "Burst spike numbers encode the amplitude of light intensity modulation" in Results. The calculation is detailed in the section "Projection onto 3-BTA−1-BTA" in the revised Materials and Methods.

Fig 5 G-I is only treated after fig 6 in the main text, this is a bit confusing.

We have changed the organization of the figures so that original Fig 5G-I are now Fig 7 in the revised manuscript.

On p. 10 is states 'litter' instead of 'little'.

We have corrected the typo.

In addition to the requested revisions, we have corrected two typos in the section "Mutual information" in Materials and Methods.

Given that the authors perform a bias correction (or that they explain why they would not), I would recommend it for publications after a few minor text edits.

We thank the reviewer again for the numerous thoughtful suggestions.
